# High diagnostic yield in skeletal ciliopathies using massively parallel genome sequencing, structural variant screening and RNA analyses

**DOI:** 10.1038/s10038-021-00925-x

**Published:** 2021-04-20

**Authors:** Anna Hammarsjö, Maria Pettersson, David Chitayat, Atsuhiko Handa, Britt-Marie Anderlid, Marco Bartocci, Donald Basel, Dominyka Batkovskyte, Ana Beleza-Meireles, Peter Conner, Jesper Eisfeldt, Katta M. Girisha, Brian Hon-Yin Chung, Eva Horemuzova, Hironobu Hyodo, Liene Korņejeva, Kristina Lagerstedt-Robinson, Angela E. Lin, Måns Magnusson, Shahida Moosa, Shalini S. Nayak, Daniel Nilsson, Hirofumi Ohashi, Naoko Ohashi-Fukuda, Henrik Stranneheim, Fulya Taylan, Rasa Traberg, Ulrika Voss, Valtteri Wirta, Ann Nordgren, Gen Nishimura, Anna Lindstrand, Giedre Grigelioniene

**Affiliations:** 1 Department of Molecular Medicine and Surgery, Center for Molecular Medicine, Karolinska Institutet, and Department of Clinical Genetics, Karolinska University Laboratory, Karolinska University Hospital, Stockholm, Sweden; 2 Division of Clinical and Metabolic Genetics, The Hospital for Sick Children, and Mt. Sinai Hospital, Toronto, ON Canada; 3 The Prenatal Diagnosis and Medical Genetics Program, Department of Obstetrics and Gynecology, Mount Sinai Hospital, University of Toronto, Toronto, ON Canada; 4 Department of Radiology, University of Iowa Hospitals and Clinics, Iowa City, IA USA; 5 Department of Women’s and Children’s Health, Neonatology, Karolinska Institutet, Stockholm, Sweden; 6 Division of Medical Genetics, Medical College of Wisconsin, Milwaukee, WI USA; 7 Department of Molecular Medicine and Surgery, Center for Molecular Medicine, Karolinska Institutet, Stockholm, Sweden; 8 Department of Clinical Genetics, University Hospitals Bristol and Weston NHS Foundation Trust, Bristol, UK; 9 Department of Women’s and Children’s Health, Karolinska Institutet and Center for Fetal Medicine, Karolinska University Hospital, Stockholm, Sweden; 10 Science for Life Laboratory, Department of Molecular Medicine and Surgery, Karolinska Institutet, and Department of Clinical Genetics, Karolinska University Laboratory, Karolinska University Hospital, Stockholm, Sweden; 11 Department of Medical Genetics, Kasturba Medical College, Manipal Academy of Higher Education, Manipal, India; 12 Department of Pediatrics and Adolescent Medicine, The University of Hong Kong and Shenzhen Hospital, Futian District, Shenzhen, China; 13 Department of Pediatrics and Adolescent Medicine, Queen Mary Hospital, The University of Hong Kong, Hong Kong, China; 14 Department of Women’s and Children’s Health, Karolinska Institutet and Paediatric Endocrinology Unit, Karolinska University Hospital, Stockholm, Sweden; 15 Department of Obstetrics and Gynecology, Tokyo Metropolitan Bokutoh Hospital, Kotobashi, Sumida-ku, Tokyo, Japan; 16 Department of Prenatal Diagnostics, Riga Maternity Hospital, Riga, Latvia; 17 Medical Genetics, MassGeneral Hospital for Children, Boston, MA USA; 18 Department of Molecular Medicine and Surgery, Karolinska Institutet, and Centre for Inherited Metabolic Diseases, Karolinska University Laboratory, Karolinska University Hospital, Stockholm, Sweden; 19 Science for Life Laboratory, School of Engineering Sciences in Chemistry, Biotechnology and Health, KTH Royal Institute of Technology, Stockholm, Sweden; 20 Medical Genetics, Tygerberg Hospital and Division of Molecular Biology and Human Genetics, Faculty of Medicine and Health Sciences, Stellenbosch University, Tygerberg, South Africa; 21 Division of Medical Genetics, Saitama Children’s Medical Center, Saitama, Japan; 22 Department of Microbiology, Tumor and Cell biology, Science for Life Laboratory, Karolinska Institutet, Stockholm, Sweden; 23 Department of Genetics and Molecular Medicine, Lithuanian University of Health Sciences, Kaunas, Lithuania; 24 Department of Pediatric Radiology, Karolinska University Hospital, Stockholm, Sweden; 25 Department of Pediatric Imaging, Tokyo Metropolitan Children’s Medical Center, Tokyo, Japan

**Keywords:** Clinical genetics, Medical genetics, Genetics research

## Abstract

Skeletal ciliopathies are a heterogenous group of disorders with overlapping clinical and radiographic features including bone dysplasia and internal abnormalities. To date, pathogenic variants in at least 30 genes, coding for different structural cilia proteins, are reported to cause skeletal ciliopathies. Here, we summarize genetic and phenotypic features of 34 affected individuals from 29 families with skeletal ciliopathies. Molecular diagnostic testing was performed using massively parallel sequencing (MPS) in combination with copy number variant (CNV) analyses and in silico filtering for variants in known skeletal ciliopathy genes. We identified biallelic disease-causing variants in seven genes: *DYNC2H1*, *KIAA0753, WDR19*, *C2CD3*, *TTC21B*, *EVC*, and *EVC2*. Four variants located in non-canonical splice sites of *DYNC2H1*, *EVC*, and *KIAA0753* led to aberrant splicing that was shown by sequencing of cDNA. Furthermore, CNV analyses showed an intragenic deletion of *DYNC2H1* in one individual and a 6.7 Mb de novo deletion on chromosome 1q24q25 in another. In five unsolved cases, MPS was performed in family setting. In one proband we identified a de novo variant in *PRKACA* and in another we found a homozygous intragenic deletion of *IFT74*, removing the first coding exon and leading to expression of a shorter message predicted to result in loss of 40 amino acids at the N-terminus. These findings establish *IFT74* as a new skeletal ciliopathy gene. In conclusion, combined single nucleotide variant, CNV and cDNA analyses lead to a high yield of genetic diagnoses (90%) in a cohort of patients with skeletal ciliopathies.

## Introduction

Ciliopathies are disorders that arise from the dysfunction of motile and/or non-motile cilia. They have a wide range of clinical manifestations, involving almost every organ, due to the key role of cilia in organogenesis [1, 2]. Skeletal ciliopathies are classified according to their radiographic features. The most common clinical entities are short-rib polydactyly syndromes (SRPS) and asphyxiating thoracic dystrophies (ATD; Jeune) [3]. In Online Mendelian Inheritance in Man (OMIM) database they are named as a broader group of short-rib thoracic dysplasias (SRTD [MIM:208500]). Less common are Ellis-van Creveld syndrome (EVC [MIM:225500]) and Sensenbrenner syndrome (also known as cranioectodermal dysplasia; CED [MIM:218330]). The most distinctive skeletal features are narrow thorax due to short, horizontally oriented ribs, short tubular bones with metaphyseal irregularities, hypoplastic pelvis with characteristic trident acetabulum and/or brachydactyly with cone-shaped epiphyses. Postaxial polydactyly may be present. Non-skeletal manifestations include nephronophthisis, renal cystic dysplasia, retinopathy, and malformations of the central nervous system, heart and genitalia. The severity of skeletal ciliopathies ranges from mild to lethal [4].

Cilia are important for cell migration and signaling in tissue development and organogenesis, including the skeleton. The ciliary proteins are synthesized in the cytoplasm and transported into the cilia in a process called intraflagellar transport (IFT) [1]. Nearly all genes associated with skeletal ciliopathies encode proteins of the IFT machinery or those important for cilia structure and function and most of the pathogenic variants are inherited in an autosomal recessive manner. In many genes associated with skeletal ciliopathies, disease-causing variants may result in other ciliopathy syndromes with a phenotypic overlap among them [2]. Recent studies have unraveled several genes associated with skeletal ciliopathies [5–14]. Massively parallel sequencing (MPS) leads to quick identification of disease-causing variants in known disease genes and the discovery of novel genes [8, 15].

In this study, we report disease-causing variants in nine ciliopathy genes and show that MPS with copy number variant (CNV) screening and cDNA sequencing lead to a high diagnostic yield of 90%. We present detailed clinical and phenotypic features of the patients in this cohort. Furthermore, we describe a homozygous intragenic deletion of *IFT74* in a newborn with ATD, adding *IFT74* to the skeletal ciliopathy gene list.

## Material and methods

### Patient cohort, clinical data and samples

This study was approved by the ethical review board at Karolinska Institutet (2012/2106-31/4, 2013/1325-31/2; 2014/983-31/1; 2018/2207-32) and informed consent from the parents or legal guardians was provided in all cases. Suspected disease-causing variants are reported in ClinVar (https://www.ncbi.nlm.nih.gov/clinvar) (ClinVar submission IDs: SCV000788357- SCV000788396, SCV000924632- SCV000924635 and SCV001438035).

Twenty-nine probands with clinically and radiologically diagnosed skeletal ciliopathies, according to the criteria described by Spranger et al. [16] were included in the study (diagnostic criteria are listed in Supplementary Table 1). Clinical data included family history, clinical features, skeletal radiographs, and autopsy reports from terminated pregnancies. The radiographic manifestations of all patients involved in this study were reviewed by three experienced radiologists (authors AtH, UV, and GN). Clinical data from patient records were retrospectively collected from the referring clinicians. Genomic DNA was extracted from peripheral blood obtained from the affected individuals and their parents or from frozen tissue samples of terminated pregnancies using standard protocols. RNA was extracted from frozen fetal lung tissue (proband 10) and from blood samples (proband 11, affected sibling of proband 14 and their parents, proband 20 and mother of proband 22) using RNeasy mini kit (Qiagen, Hilden, Germany) or PAXgene Blood RNA kit (PreAnalytiX, Hombrechtikon, Switzerland). cDNA was synthesized using RevertAid First strand synthesis kit (ThermoFischer Scientific, Waltham, MA, USA) or High capacity cDNA Reverse transcription kit (Applied Biosystems, Foster City, CA, USA) according to the supplier’s protocols. Anonymized age-matched cDNA from control samples were obtained from the biobank at Clinical Genetics, Karolinska University Hospital, Stockholm.

### Genetic analyses

**Fig. 1:**
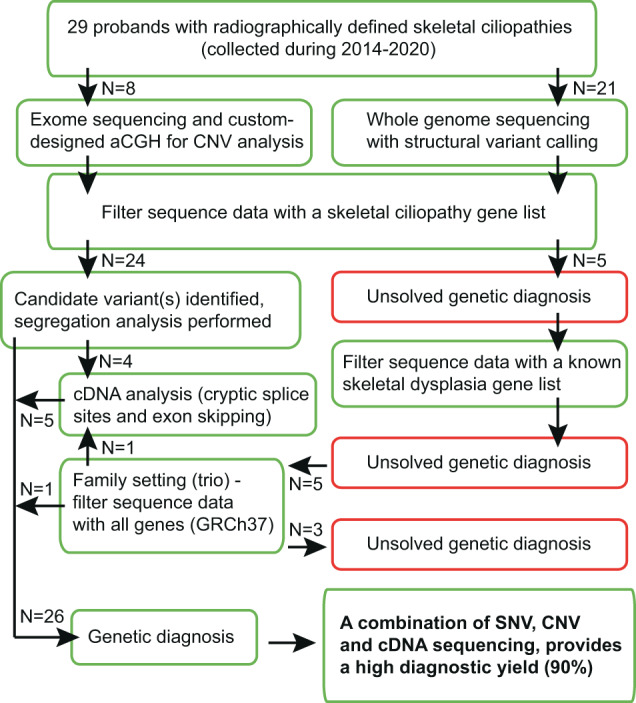
Flowchart of the study design. N the number of probands in each part of the study, aCGH array Comparative Genomic Hybridization, CNV Copy Number Variation, SNV Single Nucleotide Variant

This study is part of a project improving molecular diagnostics at the Department of Clinical Genetics, Karolinska University Hospital. When starting the project in 2014, a combination of exome sequencing and array comparative genomic hybridization (aCGH) analyses were standard methods. During 2015, we switched to whole genome sequencing (WGS) as our main method and therefore the WGS data is assessed for both single nucleotide variants (SNVs) and gene dose/structural abnormalities. For an overview of the study flow, see Fig. 1.

Sequencing and variant calling were performed as previously described [12, 17–19]. DNA samples from eight patients underwent clinical exome sequencing and from 21 patients – clinical whole genome sequencing (WGS). First, variants were ranked according to expected disease-causing potential in known skeletal ciliopathy genes (Supplementary Table 2), present in coding exons or ±20 base pairs intronic sequence and a minor allele frequency (MAF) of <0.005 according to public databases. Second, for probands with unsolved diagnoses the analysis was extended, and data filtered for known skeletal dysplasia genes according to the Nosology and Classification of Congenital Skeletal Disorders [3]. In addition, CNV analyses with exon resolution was performed for all patients. The eight samples analyzed with exome sequencing were subjected to custom-designed targeted array comparative genomic hybridization (aCGH) [19]. Structural variants from the WGS data were called using an in-house developed pipeline [18]. The breakpoints of the structural abnormalities in *DYNC2H1* and *IFT74* were confirmed with Sanger sequencing. Sanger sequencing was also used for segregation analysis in all but family 21, from which parental samples were not available. In five families (10-11, 14, 20, and 22) cDNA synthesized from RNA of the affected individuals and/or their heterozygous family members were sequenced to confirm splicing abnormalities caused by pathogenic variants in the cryptic splice sites e.g. those not located in close proximity to the exon-intron boundaries (±2 base pairs). For five individuals, whose diagnoses were unsolved in singleton analyses, we performed WGS in a family setting as trios (families 20, 24 and 27-29) and all genes (annotated in GRCh37) were analyzed.

All the individuals or their legal representatives have signed informed consents to participate in the study. However, according to European law, the General Data Protection Regulation (https://eur-lex.europa.eu/eli/reg/2016/679/oj), an individual genome data is considered to be personal, and cannot be shared as a whole data set. We can share small subsets of variants of interest upon reasonable request. Detailed information of the bioinformatic platforms and statistics is provided in the Supplementary Methods and Supplementary Table 3.

## Results

### Clinical features

The study includes 34 patients from 29 unrelated families: 26 patients from 22 families with diagnoses of SRPS and ATD, three patients from two families with CED, three patients from three families with EVC and two patients from two families with unclassified skeletal ciliopathies. Clinical features of the probands and their affected siblings are summarized in Table 1, radiographic features in Fig. 2D–I, Fig. 3B–D, Supplementary Figs. 1-25 and Supplementary Table 4. Clinical features of patient 21 have been reported previously [20], but the molecular diagnosis was not known at the time.

**Table 1:** Clinical features of the probands and their affected siblings

Family	Age & sex	Diagnosis	DD	Additional information
1	† 2d F	ATD	na	Respiratory insufficiency at birth, sibling [1b] with ATD († 8d M)
2	TOP M	ATD	na	GA 19, syndactyly of the hands
3	TOP M	SRPS3	na	GA 22, NPHP, hydrops, scoliosis, lumbar lordosis, ambiguous genitalia, oligohydramnios, joint contractures, low set posteriorly rotated ears, hypertelorism, depressed nasal bridge, midface hypoplasia
4	2 y M	ATD	−	Respiratory insufficiency at birth
5	6 y M	ATD	−	
6	TOP M	ATD	na	GA 28
7	TOP F	ATD	na	GA 19 + 3, *Coxa vara*
8	TOP M	SRPS3	na	GA 18, syndactyly, ambiguous genitalia, microcystic kidney, hypoplasia of urinary bladder, frontal bossing, cleft palate
9	TOP F	SRPS3	na	GA 21, pancreatic cysts, NPHP, low set posteriorly rotated ears
10	TOP M	ATD	na	GA 19
11	3 y M	ATD	na	Flat nasal bridge, small teeth, oligohydramnios
12	3 y M	ATD	–	Respiratory insufficiency at birth, sibling [12b] with ATD († 7d M)
13	TOP M	SRPS3	na	GA 20 + 6, hypoplasia of corpus callosum, ambiguous genitalia, malrotation of bowel, retrognathia, small tongue, low set ears
14	† 2 d M	ATD + JBTS	na	MTS, sibling [14b] with ATD and JBTS († 4 m F)
15	† 2 m M	ATD + JBTS	na	Premature birth (GA 33 + 4), MTS, IUGR, ventriculomegaly, sibling [15b] with ATD + JBTS (TOP)
16	2 y M	ATD	−	no MTS was shown on MRI, enlarged anterior ventricles
17	7 y M	ATD	+	Retrognathia, renal insufficiency, developmental delay
18	18 y M	ATD	−	NPHP (Kidney transplant at 12 y)
19	† 2 y F	ATD	−	NPHP, dolichocephaly, speech delay
20	† 7 d M	ATD	−	Respiratory insufficiency at birth
21	† 10 m F	CED	+	Macrocephaly, dolichocephaly, sparse hair, NPHP, cystic liver (Caroli disease), previously reported [19]
22	TOP M	EVC	na	GA 21, rocker bottom feet, simian creases, syndactyly of left foot, micrognathia, low set ears, up-slanting palpebral fissures
23	17 y 3 m M	EVC	mild	Short stature, structural heart abnormality
24	4 y 4 m M	EVC	−	Sparse hair, deviating thumbs, nail hypoplasia, speech delay, structural heart abnormality, hypoplastic teeth, retrognathia
25	† 1d M	unclassifiable	na	Premature birth (GA 31), respiratory insufficiency at birth
26	5 y M	unclassifiable	mild	Brachycephaly, sparse hair, crowded teeth
27	† 1 d M	ATD	na	Respiratory insufficiency at birth
28	3 m F	ATD	na	Premature birth (GA 35)
29	† 1 d M	CED	na	Respiratory insufficiency at birth, hypertelorism, midface hypoplasia, micrognathia, sibling [29b] with CED (2y4m F)

†, deceased; +, present; –, absent

*ATD* asphyxiating thoracic dystrophy, *CED* Cranioectodermal dysplasia, *d* days, *DD* Developmental delay, *EVC* Ellis-van Creveld syndrome, *F* female, *GA* gestational age (weeks + days), *IUGR* intrauterine growth restriction, *JBTS* Joubert syndrome, *m* months, *M* male, *MTS* molar tooth sign, *na* not available, *NPHP* nephronophthisis, *TOP* termination of pregnancy, *SRPS3* short-rib polydactyly syndrome type 3, *w* weeks, *y* years

**Fig. 2:**
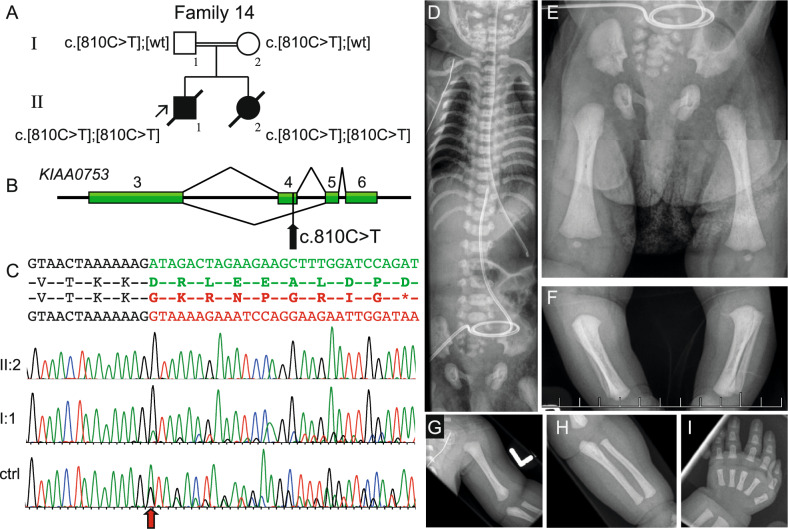
Molecular and radiographic features of the proband in family 14 with a homozygous synonymous variant introducing abnormal splicing in *KIAA0753* (NM_014804.4). **A** Pedigree of family 14 and segregation of the *KIAA0753* variant. **B** Schematic figure of the genomic region from exon 3 to 6 in *KIAA0753*, indicating the normal splice pattern (top) and abnormal splicing (bottom). The black arrow shows the position of the c.810C>T variant in exon 4. **C** Sequence of exon 3 (text in black), exon 4 (green) and exon 5 (red). cDNA sequence traces show skipping of exon 4 in homozygous state for patient II:2, heterozygous state for patients father, shown by lower peaks of normal isoform transcript in I:1 (sequence from mother I:2 not shown). Analysis of normal control sample shows that the aberrant splicing pattern in very low extent is also present in healthy individuals (*n* = 3). The red arrow shows where the exon skipping occurs. (D-I) Radiograms of II:2 at 5 days of age. Note narrow thorax, short ribs and characteristic “handle bar” appearance of the clavicles. **E**–**H** short craniocaudal diameter of the iliac bones, trident pelvis, short ischial bones and short tubular bones with bulbous ends. The hand shows short metacarpals and phalanges, note that middle phalanges are particularly short and broad (**I**). ctrl control, wt wildtype

**Fig. 3:**
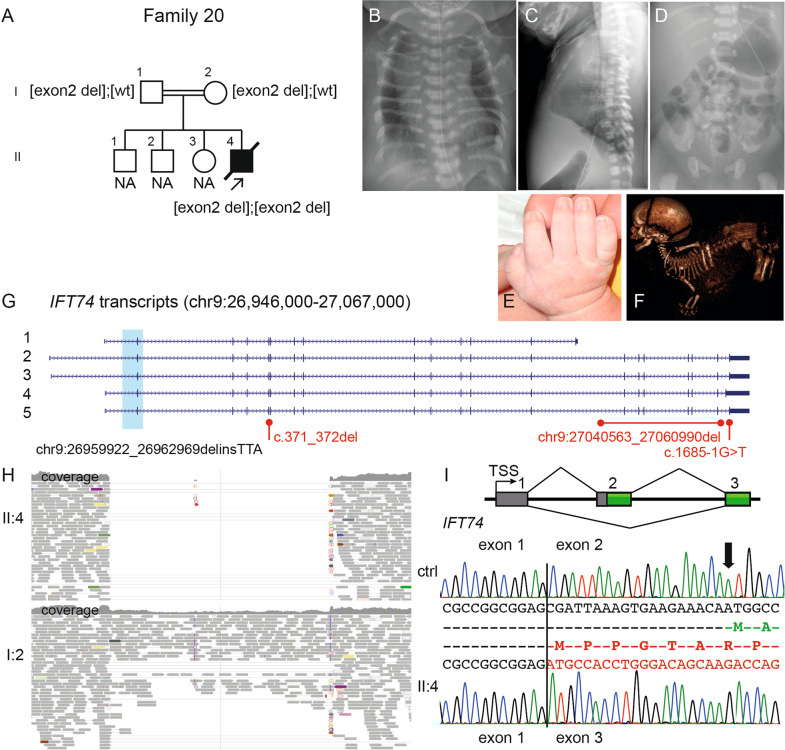
Molecular and radiographic findings of the proband in family 20 with an intragenic deletion of exon 2 in* IFT74* (NM_025103.3). **A** Pedigree of family 20. **B**–**D** Radiograms of II:4, please note **B** handle bar appearance of the clavicles; **C**–**D** narrow thorax due to short ribs and pelvis with short craniocaudal diameter of the iliac bones, medial acetabular spur and short ischial bones. **E** The hand shows mild brachydactyly and no polydactyly. **F** Fetal CT at GA 33 + 0 shows dolichocephaly, short ribs, short tubular bones and a “star-gazing” position. **G** Schematic figure of *IFT74* transcripts (NCBI RefSeq genes; 1: NM_001099224; 2: NM_001099223; 3: NM_001099222; 4: NM_001349928; 5: NM_025103) shows the deletion in light blue and variant details in black (NC_000009.11:g.26959922_26962969delinsTTA) encompassing the initiation site for all transcripts. Previously reported variants in Bardet–Biedl syndrome are marked in red. **H** IGV pileup over the genomic region with the deletion (homozygous II:4 in upper part and heterozygous parent I:2 lower part) with absent/decreased coverage and paired-end reads. **I** Schematic presentation of exons 1 to 3 in *IFT74* and cDNA sequence from age-matched control (top) shows normal splicing pattern over exon 1 and 2 (amino acids in green) and cDNA sequence from affected child (bottom) shows homozygous skipping of exon 2 (amino acids in red), leading to a new initiation site (ATG + 1) in exon 3 (in-frame). CT computer tomography, GA gestational age, IGV Integrative Genomics viewer

### Molecular findings

We identified likely disease-causing variants in 26 out of 29 probands, distributed in both previously known skeletal ciliopathy genes (*DYNC2H1*, *WDR19*, *C2CD3*, *KIAA0753, TTC21B*, *EVC*, and *EVC2*), as well as in *IFT74*, a novel skeletal ciliopathy gene. In addition, a heterozygous de novo missense variant in *PRKACA* was identified in one affected individual. A proband with clinically unclassifiable skeletal ciliopathy was diagnosed with 1q24q25 microdeletion syndrome, giving a total diagnostic yield in this study cohort of 90%. Disease-causing variants in DYNC2H1 were most common (44%). The variants are summarized in Table 2 and Supplementary Table 5.

**Table 2:** Disease-causing variants and CNVs identified in the skeletal ciliopathy cohort

Family	Gene	Nucleotide	Amino acid/mRNA	Inheritance	Previously reported	Genomic coordinates	MAF^#^
*Patients with clinical diagnoses of SRPS and ATD*							
1	*DYNC2H1*	c.7919 T > C	p.(Ile2640Thr)	P	[41]	chr11:g.103070036	–
		c.9865 G > A	p.(Asp3289Asn)	M	[8]	chr11:g.103114446	0.000003
		c.12602 C > T	p.(Ser4201Phe)	M	VCV000558738.1 ClinVar	chr11:g.103327017	0.000169
2	*DYNC2H1*	c.8003 T > G	p.(Val2668Gly)	P	–	chr11:g.103070120	0.000009
		c.9044 A > G	p.(Asp3015Gly)	M	[42]	chr11:g.103091449	0.000774
3	*DYNC2H1*	c.6910 G > A	p.(Ala2304Thr)	P	[8]	chr11:g.103058085	0.000697
		c.8444 G > A	p.(Ser2815Asn)	M	–	chr11:g.103075683	–
4	*DYNC2H1*	c.5971 A > T	p.(Met1991Leu)	M/P	[42]	chr11:g.103048381	–
		c.11284 A > G	p.(Met3762Val)	M/P	[42]	chr11:g.103175330	0.003237
5	*DYNC2H1*	c.5682_5683del	p.(His1896Tyrfs*9)	M	[43]	chr11:g.103046971-2	0.000179
		c.5771 A > T	p.(Asp1924Val)	P	–	chr11:g.103047060	–
6	*DYNC2H1*	c.729 T > A	p.(Tyr243*)	P	–	chr11:g.102987406	–
		c.9044 A > G	p.(Asp3015Gly)	M	[42]	chr11:g.103091449	0.000774
7	*DYNC2H1*	c.1855C > T	p.(Gln619*)	P	–	chr11:g.102996022	0.000105
		c.9044 A > G	p.(Asp3015Gly)	M	[42]	chr11:g.103091449	0.000774
8	*DYNC2H1*	c.2386del	p.(Arg796Glyfs*8)	M	–	chr11:g.103006489	0.000018
		c.10163 C > T	p.(Pro3388Leu)	P	VCV000439631.4 ClinVar	chr11:g.103124113	0.000140
9	*DYNC2H1*	c.624_625delGTinsAA	p.(Phe209Ile)	P	[44]	chr11:g.102987301-2	0.000157
		c.2574 + 1 G > A	p.(?)	M	–	chr11:g.103006678	–
10	*DYNC2H1*	c.7129 T > G	p.(Phe2377Val)	M	–	chr11:g.103058304	–
		c.6478-16 G > A	r.6477_6478ins6478-14_6478-1	P	–	chr11:g.103055609	0.000018
11	*DYNC2H1*	c.1306 G > T	p.(Glu436*)	P	[22]	chr11:g.102991711	–
		c.11070 G > A	r.11044_11116del	M	–	chr11:g.103158288	0.000033
12	*DYNC2H1*	c.1366 C > T	p.(Arg456*)	P	–	chr11:g.102992106	0.000059
		c.3455 T > C	p.(Phe1152Ser)	M	–	chr11:g.103025332	–
		c.5690 T > C	p.(Ile1897Thr)	M	VCV000864089.1 ClinVar	chr11:g.103046979	0.000175
		c.8354 C > A	p.(Ala2785Glu)	M	–	chr11:g.103075593	0.000076
13	*C2CD3*	c.5227 G > T	p.(Gly1743Cys)	P	[36]	chr11:g.73760516	0.000163
		c.5267 G > A	p.(Gly1756Glu)	M	[35]	chr11:g.73760476	0.000062
14	*KIAA0753*	c.810 C > T	r.719_825del	M/P	–	chr17:g.6528090	0.000296
15	*KIAA0753*	c.810 C > T	r.719_825del	M/P	–	chr17:g.6528090	0.000296
16	*KIAA0753*	c.970 C > T	p.(Arg324*)	M/P	[12]	chr17:g.6526336	0.000033
17	*TTC21B*	c.2758-2 A > G	p.(?)	M	[12, 39]	chr2:g.166756392	0.000039
		c.3857 T > C	p.(Ile1286Thr)	P	–	chr2:g.166732691	0.000028
18	*WDR19*	c.56 T > G	p.(Phe19Cys)	M	–	chr4:g.39187395	0.000029
		c.3868_3871del	p.(Thr1290Cysfs*14)	P	–	chr4:g.39279778-81	0.000019
19	*WDR19*	c.974 T > C	p.(Leu325Ser)	P	–	chr4:g.39217473	–
		c.3758 G > A	p.(Cys1253Tyr)	M	–	chr4:g.39278681	0.000065
20	*IFT74*	NC_000009.11: g.26959922_26962969delinsTTA	r.-19_120del	M/P	–	–	–
*Patient with a clinical diagnosis of CED*							
21	*WDR19*	c.1623C > G	p.(Tyr541*)	NA	–	chr4:g.39226647	0.000043
		c.3533 G > A	p.(Arg1178Gln)	NA	[45]	chr4:g.39274649	0.000388
*Patients with clinical diagnoses of EVC*							
22	*EVC*	c.1018 C > T	p.(Arg340*)	P	[46]	chr4:g.5749953	0.000138
		c.175-9 G > A	r.174_175ins175-7_175-1	M	–	chr4:g.5720966	0.000062
23	*EVC2*	c.571 A > G	p.(Asn191Asp)	M/P	–	chr4:g.5691019	–
24	*PRKACA*	c.409 G > A	p.(Gly137Arg)	de novo	[14]	chr19:g.14211648	–
*Patients with unclassified skeletal ciliopathies*							
25	*DYNC2H1*	c.10022 C > G	p.(Pro3341Arg)	P	–	chr11:g.103116062	–
		NC_000011.10: g.103016481_103177263del	p.(Ser901Argfs*1?)	M	–	–	–
26	*–*	NC_000001.11: g.169095250_175778910del	1q24q25 deletion	de novo	[40]	–	–

–, absent; #, gnomAD v2.1.1 and gnomAD v2 SVs

Reference sequences and genomic coordinates according to hg19 [GRCh37]: *C2CD3* (NM_015531.5), *DYNC2H1* (NM_001080463.1), *EVC* (NM_001306090.1), *EVC2* (NM_147127.4), *IFT74* (NM_025103.2), *KIAA0753* (NM_014804.2), *PRKACA* (NM_002730.4); *TTC21B* (NM_024753.5), *WDR19* (NM_025132.3)

*ATD* asphyxiating thoracic dystrophy, *CED* cranioectodermal dysplasia, *chr* chromosome, *EVC* Ellis-van Creveld syndrome, *MAF* minor allele frequency (Popmax), *M* maternal, *M/P* homozygous variant, *NA* not available, *P* paternal, *SRPS* short-rib polydactyly syndrome

#### Synonymous variants leading to exon skipping

In family 14, two deceased children from a consanguineous family (Fig. 2A), had overlapping features with ATD and Joubert syndrome (JBTS [MIM:213300]). Skeletal surveys of both children showed narrow thorax, short ribs, trident pelvis and metaphyseal flaring and MRI showed molar tooth sign (MTS) (Supplementary Fig. 26). WGS of all known skeletal ciliopathy and dysplasia genes did not reveal any known disease-causing variants, but a very rare homozygous synonymous variant in *KIAA0753* (c.810 C > T, p.=) was identified. This variant segregated in the family, raising the hypothesis that it may affect splicing. Sequencing of the cDNA from affected sibling (II:2) to proband 14 and their parents (I:1 and I:2) showed that the variant leads to skipping of exon 4 (r.719_825del) and a premature stop codon (p.Asp240Glyfs*8) (Fig. 2B–C), which is predicted to result in nonsense mediated decay (NMD). Analysis of parental cDNA showed relative abundance of the exon-skipping transcript. The aberrant transcript is reported in Ensembl (ENST00000570790.1), but the normal isoform predominates in age-matched controls (Fig. 2C). The RNA extracted from the blood samples from the family was not sufficient for subsequent qPCR analysis. The same homozygous variant was found in the proband from family 15, who deceased at two months of age. No RNA sample was available from this individual. Both families were of Pakistani origin, but according to the family history they were not related. These families share a homozygous block of 85 kb on chromosome 17p13.1, including the *KIAA0753* gene, indicating a common ancestor.

**Fig. 4:**
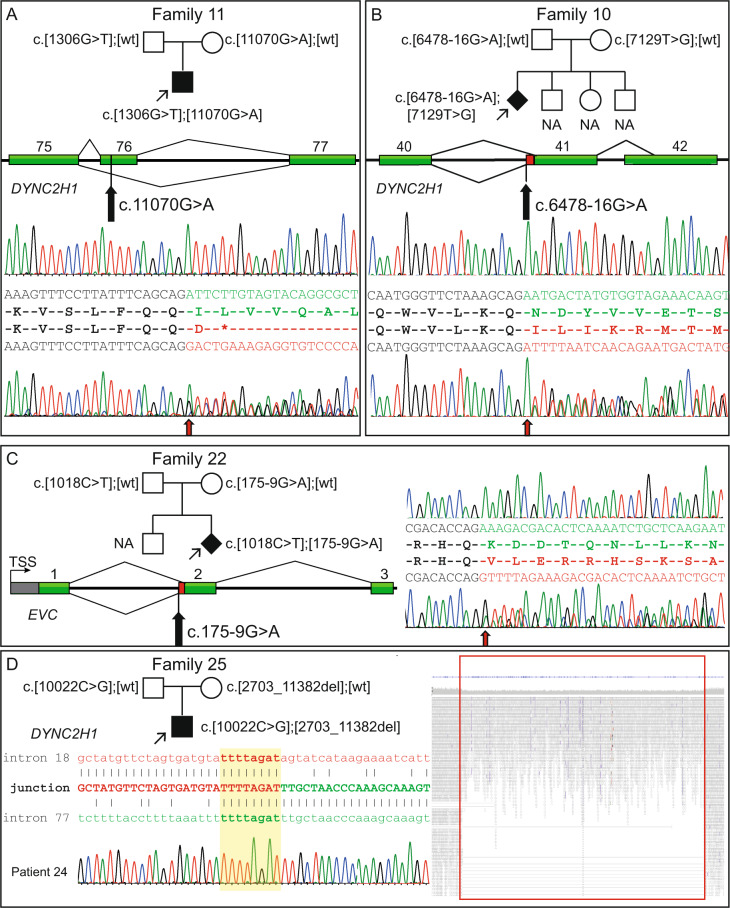
Molecular features of splice variants in *DYNC2H1* (NM_001080463.1), *EVC* (NM_001306090.1) and an intragenic deletion in *DYNC2H1* and pedigrees of the affected families. **A** Family 11 showing segregation for the variants in *DYNC2H1* and a schematic presentation of exons 75 to 77 of the gene. cDNA sequence from normal control individual (top) shows normal splicing pattern of exon 75 and 76 (in green), and from affected child (bottom) shows heterozygous skipping of exon 76 (in red), leading to a premature stop codon. At the start of the frameshift (red arrow) sequences of both exon 76 and 77 are visible. **B** Family 10 showing the segregation of the variants in *DYNC2H1* and a schematic presentation of exons 40 to 42 of the gene. The red bar indicates extra bases added to the gene product due to the introduced cryptic splice site variant. cDNA sequence from a normal control individual (top) shows normal splicing (in green) at the exon-intron boundary of exon 41. Sequencing of the same fragment from the affected fetus (bottom) shows an insertion of 14 bp to the cDNA (in red) leading to frameshift and a premature stop. Red arrow shows start of exon 41. **C** Family 22 showing segregation of the variants in *EVC* (NM_001306090.1) and a schematic presentation of the beginning of the gene. The red bar indicates extra bases added to the gene product when a cryptic splice site variant is inserted. cDNA sequence from a control individual (top) shows normal splicing at the exon-intron boundary of exon 2 (in green). Sequencing of the same fragment from the mother (bottom), who is a heterozygous carrier of the variant, shows an insertion of 7 bp to the cDNA (in red) leading to a frameshift. Red arrow shows start of exon 2. **D** Pedigree of family 25 showing the segregation of the variants in *DYNC2H1* and a schematic presentation of the breakpoint junction between intron 18 and 77. Highlighted in yellow is the 8 bp microhomology and Sanger sequence trace from the affected child over the breakpoint. Reads and coverage of genome data in the deleted region from the affected child shows split reads and the drop in coverage over the intragenic deletion. bp, base pair; NA; not available; TSS, transcription start site; wt, wildtype

Proband in family 11, with a nonsense variant in *DYNC2H1* (c.1306 G > T, p.Glu436*) inherited from father, had a synonymous rare variant c.11070 G > A p.(=), 27 nucleotides downstream of the 5’ splice site in *DYNC2H1*, inherited from mother. Since there were no other potential disease-causing variants in *DYNC2H1*, we sequenced cDNA from the patient and found that this variant led to exon skipping (r.11044_11116del, p.Ile3675Aspfs*) and a premature stop codon (Fig. 4A). In this family, we also detected a nonsense variant in *IFT43* (c.73 C > T; p.Arg25*) inherited from a healthy mother.

#### Splice events caused by activation of cryptic splice sites

Probands from families 10 and 22 had variants creating cryptic splice sites and outcompeting the nearby located canonical splice site. For proband from family 10, only one heterozygous missense variant in *DYNC2H1* (c.7129 T > G, p.Phe2377Val) was identified by Sanger sequencing 10 years ago, and the molecular diagnosis remained unsolved until the identification of the cryptic splice site. With exome sequencing we also detected a rare intronic variant in *DYNC2H1*, c.6478-16 G > A. Prediction tools indicated introduction of a new splice site (Supplementary Fig. 27C). Sequencing of cDNA confirmed addition of 14 nucleotides to exon 41 (r.6477_6478ins6478-14_6478-1), leading to a frameshift (p.Asn2160Ilefs*8) (Fig. 4B). Proband in family 22 was compound heterozygous for a nonsense variant in *EVC* (c.1018 C > T, p.Arg340*) and a predicted cryptic splice variant (c.175-9 G > A) segregating in the family. No RNA was available from the fetus, but cDNA from peripheral blood from the heterozygous mother was sequenced. The variant introduced a cryptic splice site, adding seven nucleotides to exon 2 (r.174_175ins175-7_175-1) resulting in a frameshift (p.Lys59Valfs*14) with predicted NMD (Fig. 4C).

#### Structural variants

In family 20, the fourth child of a consanguineous couple died at one week of age due to thoracic hypoplasia and respiratory insufficiency. The singleton WGS analysis (performed on fetal DNA obtained from amniotic fluid) was unsolved since we only analyzed a gene panel for known skeletal dysplasia genes. Analyzing WGS data in a family setting showed a homozygous deletion of 3 kb including exon 2 of *IFT74* (NC_000009.11:g.26959922_26962969delinsTAA). Breakpoint PCR confirms the deletion (Supplementary Fig. 28). Exon 2 contains initiation site for all transcripts of *IFT74* (Fig. 3G). Sequencing cDNA from the affected child showed absence of exon 2 and a new initiation site at first base of exon 3 (Fig. 3I). The deletion includes 19 nucleotides of the 5ʹ-UTR and 40 amino acids at the N-terminus.

WGS from proband in family 25 showed a hemizygous missense variant in *DYNC2H1*, c.10022 C > G, p.(Pro3341Arg) in compound with a 161 kb intragenic deletion including exons 19 to 77 (NC_000011.10:g.103016481_103177263del). The variants segregated in the family and breakpoint-PCR using genomic DNA confirmed the deletion (Fig. 4D).

Patient 26, with a mild skeletal ciliopathy phenotype and no sequence abnormalities in known ciliopathy and skeletal dysplasia genes was found to have a 6.7 Mb heterozygous de novo deletion on chromosome 1 (1q24q25 microdeletion syndrome).

#### Trio sequencing of genetically unsolved patients

Among the tested patients five did not reveal any disease-causing variants in known skeletal dysplasia genes. Parental samples were added and re-analyzed as trios (for all known human genes) using WGS. One of the individuals were heterozygous for previously reported variant in *IFT122* [21] and the other two had one likely pathogenic variant in known skeletal cilia genes (*DYNC2H1* and *KIAA0856*, respectively), but no plausible compound candidate variants were identified (candidate variants from the trio analyses are listed in Supplementary Table 6). One patient presented with a homozygous intragenic deletion of *IFT74* as described above and the last patient had a de novo variant in *PRKACA* (NM_002730.4) c.409 G > A, p.(Gly137Arg). The same variant was recently reported in three unrelated families initially diagnosed with either EVC or Weyers acrodental dysostosis (WAD) [14].

## Discussion

Ciliopathies comprise a genetically heterogeneous group of conditions, which may present difficulties in molecular diagnosis. The approach using MPS in combination with structural variant and RNA analyses of 29 probands with clinical phenotypes of skeletal ciliopathies led to a diagnostic yield of 90%. The most frequently mutated gene is *DYNC2H1* (44%), in agreement with previously reported cohorts of skeletal ciliopathies [8, 15, 22]. In this study we also identified a homozygous intragenic deletion of *IFT74* in a newborn with severe asphyxiating thoracic dystrophy. Pathogenic variants in *IFT74* have previously been reported in Bardet–Biedl syndrome type 20 (BBS20 [MIM:617119]) in two patients without  signs of skeletal dysplasia [23, 24]. Our hypothesis is that the homozygous deletion removes the initiation site in all *IFT74* isoforms, as well as a part of the 5ʹ UTR regulatory region (Fig. 3G). cDNA sequencing of the patient sample indicates presence of a new initiation site in the beginning of exon 3, which is predicted to lead to a loss of 40 amino acids in the N-terminus of the protein (Fig. 3I). Altogether, this would result in a complete loss of normal IFT74 products and the severe phenotype in our patient is likely due to the absence of all IFT74 isoforms. Unfortunately, no frozen tissue is available for further studies since autopsy for this patient was declined by the family. We hypothesize that one or both of the *IFT74* variants in the BBS20 patients have a hypomorphic effect, thus resulting in a non-lethal phenotype. In contrast, a complete loss of IFT components is detrimental to cilia formation and is not compatible with life [25]. IFT74 together with IFT81 are key proteins in the intraflagellar transport system [26] and pathogenic variants in *IFT81* have previously been associated with ATD [10, 19]. In the light of the known clinical overlap between different ciliopathies and their genetic heterogeneity and pleiotropy, it is not surprising that disease-causing variants in *ITF74* as well as in *IFT81* can lead to similar features.

When singleton analysis did not reveal any disease-causing variants in known skeletal dysplasia genes in proband 24 with a clinical diagnosis of EVC syndrome, we continued with trio-analysis and identified a de novo variant in *PRKACA* (c.409 G > A, p.(Gly137Arg)). The same variant was recently reported in three unrelated families, in which eight affected individuals were diagnosed with EVC syndrome or WAD. *PRKACA* codes for the Cα-subunit in protein kinase A (PKA), function of which is to prevent the transcription of hedgehog (Hh) target genes through phosphorylation of GLI transcription factors. The primary cilia have an essential role in Hh signaling and the ciliopathy-like phenotype in these patients is thought to be due to reduced hedgehog signaling [14].

Diagnoses of three patients remained unsolved in this cohort and we could only identify heterozygous candidate variants of unknown clinical significance in the known skeletal ciliopathy genes. It is possible that the disease-causing variants of these patients are in deep intronic or regulatory regions of these genes, which is challenging to interpret using the current methodology. Another possibility is that there are novel genes involved in these phenotypes and that the identified variants are perhaps adding up to the mutational load [23]. Future studies using RNA-sequencing may be helpful in identifying pathogenic variants in already known genes as suggested recently [27, 28].

When only one heterozygous disease-causing variant or a rare synonymous variant was detected initially, meticulous phenotyping led us to re-evaluation of the sequencing data. In four patients (probands in family 10, 11, 14 and 22) we then identified rare compound heterozygous variants in the corresponding genes. In silico prediction tools and the extremely low allele frequency for the variants in close proximity to the exon-intron boundaries (c.6478-16 G > A in *DYNC2H1* and c.175-9 G > A in *EVC*) suggested possibility of cryptic splice sites that outcompete the canonical sites leading to inclusion of intronic sequences into the mRNA. The other two variants were synonymous (c.11070 G > A in *DYNC2H1* and c.810 C > T in *KIAA0753*) and were not conclusive regarding prediction of the aberrant splicing with in silico tools. Regulating splicing proteins bind to both intronic and exonic recognition sites and function as enhancers or silencers of the splicing machinery, but these recognition sites are not well defined and hard to interpret at the sequence level using conventional algorithms [29]. The fact that five probands in this small cohort received molecular diagnoses due to RNA studies emphasizes the need to scrutinize sequence data when only one potentially pathogenic variant is identified in a gene that could explain the phenotype. It also underlines the importance not to underestimate rare synonymous variants and their potential splicing effects. Furthermore, this study highlights the importance of a good phenotypic description and a strong primary clinical hypothesis.

Genotype-phenotype correlation in skeletal ciliopathies is difficult [4, 30]. In total, among our patients with biallelic disease-causing variants in *DYNC2H1* only four children are alive after 2 years of age (patients 4, 5, 11 and 12). Of the remaining patients with *DYNC2H1-*related disease, four infants died (patients 1a, 1b, 12b and 25) and seven affected pregnancies were terminated. In addition, to our knowledge, *KIAA0753*-related conditions are also associated with significant risk for early lethality. Altogether, there are 13 individuals reported with pathogenic variants in *KIAA0753*, out of them two pregnancies were terminated, three patients died before three months of age, one patient deceased at seven years of age, and seven patients are to our knowledge alive [12, 31–33]. Of note, a four year old proband in family 16 with a homozygous nonsense variant in *KIAA073* has a mild skeletal dysplasia and MR signs of slightly enlarged lateral ventricles. This variant has previously been reported in homozygous state in two other families with ATD and JBTS [12]. In many cases, the course of the disease and the prognosis may depend on the severity of the thoracic hypoplasia, ciliary dysfunction or internal malformations.

In this study, two patients with radiographic phenotypes of ATD had disease-causing variants in *WDR19*, previously most often associated with CED, further confirming the broad phenotypic overlap in skeletal ciliopathies. *C2CD3* pathogenic variants were previously reported in two siblings with SRTD with features of orofaciodigital syndrome (OFD) [5], resembling patient 13 in our study, who is compound heterozygous for two missense variants in C2CD3, p.(Gly1743Cys) and p.(Gly1756Glu). Both variants affect highly evolutionarily conserved amino acids within the same domain, which is involved in protein-protein interactions important for ciliogenesis [34]. Both variants were previously reported separately in a compound heterozygous state with protein-truncating variants in a patient with OFD-like JBTS (p.(Gly1756Glu)) [35] and in two siblings with a classic JBTS phenotype (p.(Gly1743Cys)), respectively [36]. Both OFD and JBTS are clinically and genetically heterogeneous diseases with an extensive overlap with skeletal ciliopathies [7, 12, 37, 38]. We conclude that it is very likely that the variants in *C2CD3* in our patient are disease-causing. In addition, pathogenic variants in *TTC21B* have been reported in patients with SRTD with end-stage renal disease, as well as in isolated nephronophthisis [39]. In this study we also have a 7-year-old patient with ATD, renal insufficiency and compound heterozygosity for variants in *TTC21B*. Altogether, these findings are consistent with a broad clinical and molecular overlap across the ciliopathy spectrum. Interestingly, families 1, 4 and 12 have two or three rare variants in *DYNC2H1* present on the same allele and whether there is one of them causing disease or if there is a combined additive effect is still unknown. Furthermore, proband in family 26 with a mild skeletal ciliopathy phenotype had a 6.7 Mb deletion at 1q24q25. This shows the importance of CNV analysis and that 1q24q25 microdeletion syndrome is an important differential diagnosis to skeletal ciliopathies. Previous reports indicated that patients with 1q24q25 microdeletion syndrome have skeletal features with short stature, brachydactyly and underdeveloped part of lower pelvis [40], similarly as our patient, leading to a clinical suspicion of mild skeletal ciliopathy. For the previously reported patients no correlation between the phenotype severity and deletion size was found and the skeletal features were thought to be due to haploinsufficiency of a microRNA cluster (miR199-miR214) [40]. According to our knowledge there are no known cilia genes in the deletion, indicating that skeletal features of this microdeletion syndrome should be considered as a phenocopy of skeletal ciliopathies.

In summary, advances in sequencing analysis encourage efforts to re-analyze the ciliary genes in individuals without a genetic diagnosis. As shown in this study sequencing alone might not be sufficient to provide a definitive diagnosis. A combination of SNV, CNV, and cDNA sequencing, provides a high diagnostic yield (90%) in our patients with skeletal ciliopathies.

## Supplementary information

Supplementary Figures

Supplementary Methods

Supplementary Tables

## References

[1] Reiter JF, Leroux MR (2017). Genes and molecular pathways underpinning ciliopathies. Nat Rev Mol Cell Biol.

[2] Mitchison HM, Valente EM (2017). Motile and non-motile cilia in human pathology: from function to phenotypes. J Pathol.

[3] Mortier GR, Cohn DH, Cormier-Daire V, Hall C, Krakow D, Mundlos S (2019). Nosology and classification of genetic skeletal disorders: 2019 revision. Am J Med Genet Part A.

[4] Keppler-Noreuil KM, Adam MP, Welch J, Muilenburg A, Willing MC (2011). Clinical insights gained from eight new cases and review of reported cases with Jeune syndrome (asphyxiating thoracic dystrophy). Am J Med Genet Part A.

[5] Cortes CR, McInerney-Leo AM, Vogel I, Rondon Galeano MC, Leo PJ, Harris JE (2016). Mutations in human C2CD3 cause skeletal dysplasia and provide new insights into phenotypic and cellular consequences of altered C2CD3 function. Sci Rep..

[6] McInerney-Leo AM, Wheeler L, Marshall MS, Anderson LK, Zankl A, Brown MA (2017). Homozygous variant in C21orf2 in a case of Jeune syndrome with severe thoracic involvement: extending the phenotypic spectrum. Am J Med Genet Part A.

[7] Tuz K, Bachmann-Gagescu R, O’Day DR, Hua K, Isabella CR, Phelps IG (2014). Mutations in CSPP1 cause primary cilia abnormalities and Joubert syndrome with or without Jeune asphyxiating thoracic dystrophy. Am J Hum Genet.

[8] Zhang W, Taylor SP, Ennis HA, Forlenza KN, Duran I, Li B (2018). Expanding the genetic architecture and phenotypic spectrum in the skeletal ciliopathies. Hum Mutat.

[9] Paige Taylor S, Kunova Bosakova M, Varecha M, Balek L, Barta T, Trantirek L, et al. An inactivating mutation in intestinal cell kinase, ICK, impairs hedgehog signalling and causes short rib-polydactyly syndrome. Human molecular genetics. 2016. 10.1093/hmg/ddw240 PMC5291234 27466187

[10] Duran I, Taylor SP, Zhang W, Martin J, Forlenza KN, Spiro RP (2016). Destabilization of the IFT-B cilia core complex due to mutations in IFT81 causes a Spectrum of Short-Rib Polydactyly Syndrome. Sci Rep..

[11] Toriyama M, Lee C, Taylor SP, Duran I, Cohn DH, Bruel AL (2016). The ciliopathy-associated CPLANE proteins direct basal body recruitment of intraflagellar transport machinery. Nat Genet.

[12] Hammarsjo A, Wang Z, Vaz R, Taylan F, Sedghi M, Girisha KM (2017). Novel KIAA0753 mutations extend the phenotype of skeletal ciliopathies. Sci Rep..

[13] Casey JP, Brennan K, Scheidel N, McGettigan P, Lavin PT, Carter S (2016). Recessive NEK9 mutation causes a lethal skeletal dysplasia with evidence of cell cycle and ciliary defects. Hum Mol Genet.

[14] Palencia-Campos A, Aoto PC, Machal EMF, Rivera-Barahona A, Soto-Bielicka P, Bertinetti D (2020). Germline and Mosaic Variants in PRKACA and PRKACB Cause a Multiple Congenital Malformation Syndrome. Am J Hum Genet.

[15] McInerney-Leo AM, Harris JE, Leo PJ, Marshall MS, Gardiner B, Kinning E (2015). Whole exome sequencing is an efficient, sensitive and specific method for determining the genetic cause of short-rib thoracic dystrophies. Clin Genet.

[16] Spranger J, Brill PW, Hall C, Nishimura G, Superti-Furga A and Unger S Bone Dysplasias-An atlas of Genetic Disorders of Skeletal Development. 4th ed: OUP USA; 2018.

[17] Leal GF, Nishimura G, Voss U, Bertola DR, Astrom E, Svensson J, et al. Expanding the Clinical Spectrum of Phenotypes Caused by Pathogenic Variants in PLOD2. J Bone Miner Res. 2017. 10.1002/jbmr.3348 29178448

[18] Lindstrand A, Eisfeldt J, Pettersson M, Carvalho CMB, Kvarnung M, Grigelioniene G (2019). From cytogenetics to cytogenomics: whole-genome sequencing as a first-line test comprehensively captures the diverse spectrum of disease-causing genetic variation underlying intellectual disability. Genome Med.

[19] Pettersson M, Vaz R, Hammarsjo A, Eisfeldt J, Carvalho CMB, Hofmeister W (2018). Alu-Alu mediated intragenic duplications in IFT81 and MATN3 are associated with skeletal dysplasias. Hum Mutat.

[20] Lin AE, Traum AZ, Sahai I, Keppler-Noreuil K, Kukolich MK, Adam MP (2013). Sensenbrenner syndrome (Cranioectodermal dysplasia): clinical and molecular analyses of 39 patients including two new patients. Am J Med Genet Part A.

[21] Tsurusaki Y, Yonezawa R, Furuya M, Nishimura G, Pooh RK, Nakashima M (2014). Whole exome sequencing revealed biallelic IFT122 mutations in a family with CED1 and recurrent pregnancy loss. Clin Genet.

[22] Schmidts M, Arts HH, Bongers EM, Yap Z, Oud MM, Antony D (2013). Exome sequencing identifies DYNC2H1 mutations as a common cause of asphyxiating thoracic dystrophy (Jeune syndrome) without major polydactyly, renal or retinal involvement. J Med Genet.

[23] Lindstrand A, Frangakis S, Carvalho CM, Richardson EB, McFadden KA, Willer JR (2016). Copy-Number Variation Contributes to the Mutational Load of Bardet-Biedl Syndrome. Am J Hum Genet.

[24] Kleinendorst L, Alsters SIM, Abawi O, Waisfisz Q, Boon EMJ, van den Akker ELT (2020). Second case of Bardet-Biedl syndrome caused by biallelic variants in IFT74. Eur J Hum Genet: EJHG.

[25] Huangfu D, Liu A, Rakeman AS, Murcia NS, Niswander L, Anderson KV (2003). Hedgehog signalling in the mouse requires intraflagellar transport proteins. Nature.

[26] Kubo T, Brown JM, Bellve K, Craige B, Craft JM, Fogarty K (2016). Together, the IFT81 and IFT74 N-termini form the main module for intraflagellar transport of tubulin. J Cell Sci.

[27] Kremer LS, Bader DM, Mertes C, Kopajtich R, Pichler G, Iuso A (2017). Genetic diagnosis of Mendelian disorders via RNA sequencing. Nat Commun.

[28] Cummings BB, Marshall JL, Tukiainen T, Lek M, Donkervoort S, Foley AR (2017). Improving genetic diagnosis in Mendelian disease with transcriptome sequencing. Sci Transl Med.

[29] Lee Y, Rio DC (2015). Mechanisms and Regulation of Alternative Pre-mRNA Splicing. Annu Rev Biochem.

[30] Bredrup C, Saunier S, Oud MM, Fiskerstrand T, Hoischen A, Brackman D (2011). Ciliopathies with skeletal anomalies and renal insufficiency due to mutations in the IFT-A gene WDR19. Am J Hum Genet.

[31] Chevrier V, Bruel AL, Van Dam TJ, Franco B, Lo Scalzo M, Lembo F (2016). OFIP/KIAA0753 forms a complex with OFD1 and FOR20 at pericentriolar satellites and centrosomes and is mutated in one individual with oral-facial-digital syndrome. Hum Mol Genet.

[32] Stephen J, Vilboux T, Mian L, Kuptanon C, Sinclair CM, Yildirimli D (2017). Mutations in KIAA0753 cause Joubert syndrome associated with growth hormone deficiency. Hum Genet.

[33] Faudi E, Brischoux-Boucher E, Huber C, Dabudyk T, Lenoir M, Baujat G (2020). A new case of KIAA0753-related variant of Jeune asphyxiating thoracic dystrophy. Eur J Med Genet.

[34] Thauvin-Robinet C, Lee JS, Lopez E, Herranz-Perez V, Shida T, Franco B (2014). The oral-facial-digital syndrome gene C2CD3 encodes a positive regulator of centriole elongation. Nat Genet.

[35] Bachmann-Gagescu R, Dempsey JC, Phelps IG, O’Roak BJ, Knutzen DM, Rue TC (2015). Joubert syndrome: a model for untangling recessive disorders with extreme genetic heterogeneity. J Med Genet.

[36] Srour M, Hamdan FF, McKnight D, Davis E, Mandel H, Schwartzentruber J (2015). Joubert Syndrome in French Canadians and Identification of Mutations in CEP104. Am J Hum Genet.

[37] Bruel AL, Franco B, Duffourd Y, Thevenon J, Jego L, Lopez E (2017). Fifteen years of research on oral-facial-digital syndromes: from 1 to 16 causal genes. J Med Genet.

[38] Malicdan MC, Vilboux T, Stephen J, Maglic D, Mian L, Konzman D (2015). Mutations in human homologue of chicken talpid3 gene (KIAA0586) cause a hybrid ciliopathy with overlapping features of Jeune and Joubert syndromes. J Med Genet.

[39] Davis EE, Zhang Q, Liu Q, Diplas BH, Davey LM, Hartley J (2011). TTC21B contributes both causal and modifying alleles across the ciliopathy spectrum. Nat Genet.

[40] Chatron N, Haddad V, Andrieux J, Desir J, Boute O, Dieux A (2015). Refinement of genotype-phenotype correlation in 18 patients carrying a 1q24q25 deletion. Am J Med Genet A..

[41] Schmidts M, Frank V, Eisenberger T, Al Turki S, Bizet AA, Antony D (2013). Combined NGS approaches identify mutations in the intraflagellar transport gene IFT140 in skeletal ciliopathies with early progressive kidney Disease. Hum Mutat.

[42] Dagoneau N, Goulet M, Genevieve D, Sznajer Y, Martinovic J, Smithson S (2009). DYNC2H1 mutations cause asphyxiating thoracic dystrophy and short rib-polydactyly syndrome, type III. Am J Hum Genet.

[43] Okamoto T, Nagaya K, Kawata Y, Asai H, Tsuchida E, Nohara F, et al. Novel compound heterozygous mutations in DYNC2H1 in a patient with severe short-rib polydactyly syndrome type III phenotype. Congenital anomalies. 2014. 10.1111/cga.12098 25410398

[44] Merrill AE, Merriman B, Farrington-Rock C, Camacho N, Sebald ET, Funari VA (2009). Ciliary abnormalities due to defects in the retrograde transport protein DYNC2H1 in short-rib polydactyly syndrome. Am J Hum Genet.

[45] Halbritter J, Porath JD, Diaz KA, Braun DA, Kohl S, Chaki M (2013). Identification of 99 novel mutations in a worldwide cohort of 1,056 patients with a nephronophthisis-related ciliopathy. Hum Genet.

[46] Ruiz-Perez VL, Ide SE, Strom TM, Lorenz B, Wilson D, Woods K (2000). Mutations in a new gene in Ellis-van Creveld syndrome and Weyers acrodental dysostosis. Nat Genet.

