# Supplementary material for: High diagnostic yield in skeletal ciliopathies using massively parallel genome sequencing, structural variant screening and RNA analyses
Source: J Hum Genet. 2021 Apr 20;66(10):995–1008. doi: 10.1038/s10038-021-00925-x (PMC8472897; doi:10.1038/s10038-021-00925-x)
Supplement: Supplementary file 1 — Supplementary Figures [file 10038_2021_925_MOESM1_ESM.pdf]

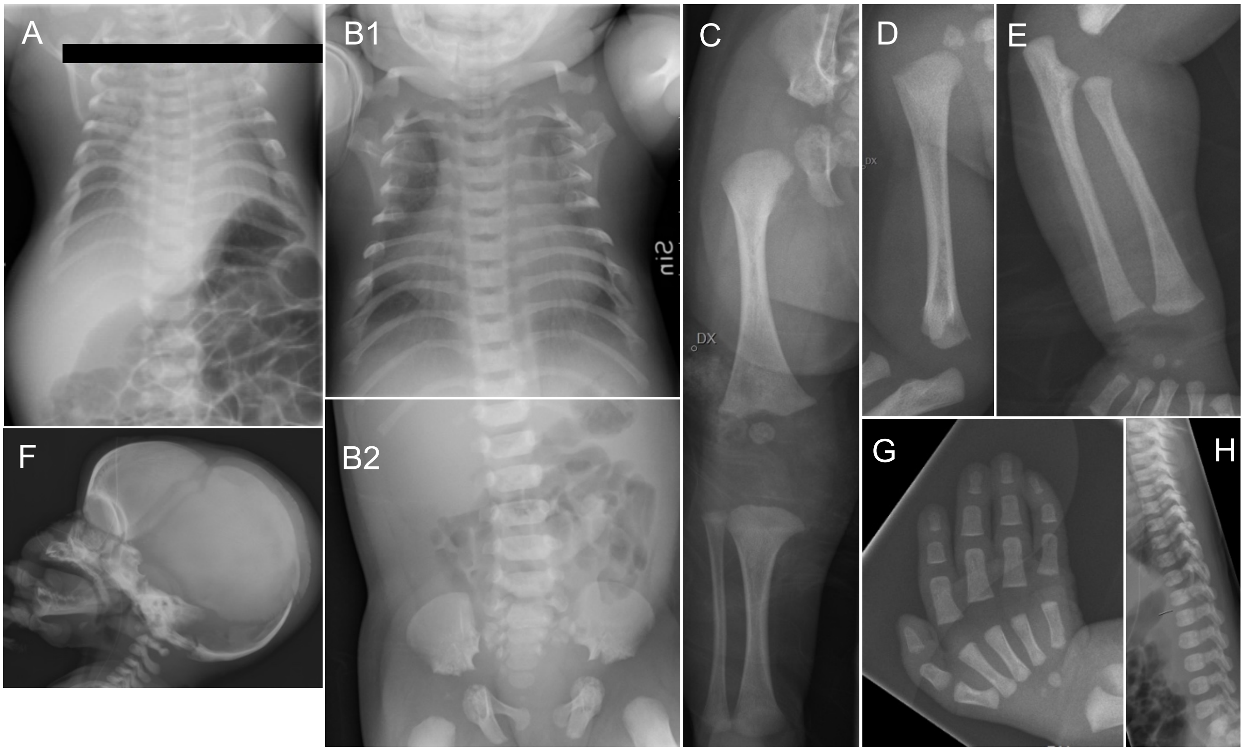

Supplementary Figure 1. Radiographs of two affected children with ATD from family 1 with pathogenic variants in *DYNC2H1*. (A) A newborn female shows a narrow thorax with short ribs. (B1-B2) A newborn male sibling shows a handlebar appearance of the clavicles, short ribs with splaying of the anterior ends and craniocaudal shortening of the ilia with trident acetabula. (C-E) Mild shortening of the long bones with flared metaphyses, especially at the knee. There are advanced ossification of the proximal humeral epiphyses and premature ossification of the right capital femoral epiphysis. (G) The metacarpals and phalanges are mildly short. Note premature carpal ossification. (F-H) Skull and spine unremarkable. *Reused S1-figures from PMID:31965514*

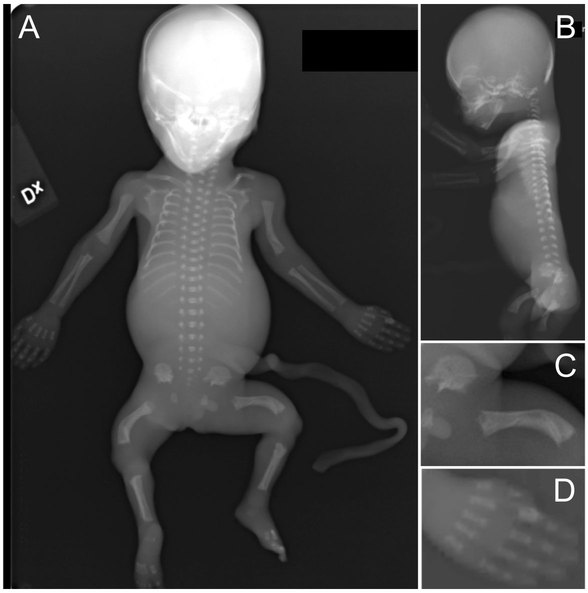

Supplementary Figure 2. Radiographs of a fetus with ATD from family 2 with pathogenic variants in *DYNC2H1*; TOP at 19 weeks GA. (A) There are short ribs, short ilia with trident acetabula, shortening of the long tubular bones with mild metaphyseal irregularities, and bowing of the femora. (B) The skull and spine are unremarkable. (C) Magnified view of the bowed femur. (D) Magnified view of the hand shows mildly short metacarpals and phalanges. (TOP, termination of pregnancy) *(S2-figures reused with permission from Japan Journal of Radiology, PMID:31965514)*

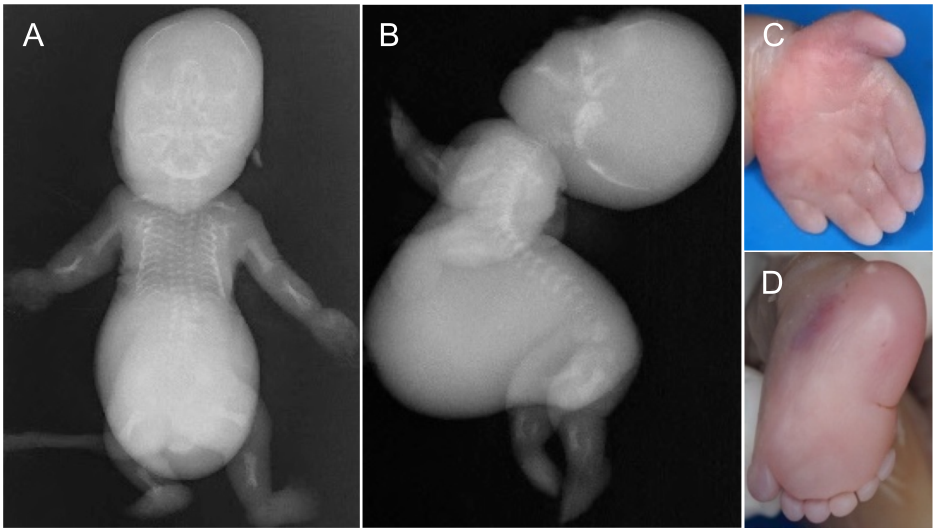

Supplementary Figure 3. Radiographs and clinical photographs of a fetus with SRPS3 from family 3 with pathogenic variants in *DYNC2H1* (TOP at 22 weeks GA). (A-B) There are short ribs, short ilia and severe shortening of the long bones with metaphyseal irregularities. The vertebral bodies are delayed in ossification. The abdomen is protuberant. (C-D) Brachydactyly and postaxial polydactyly.

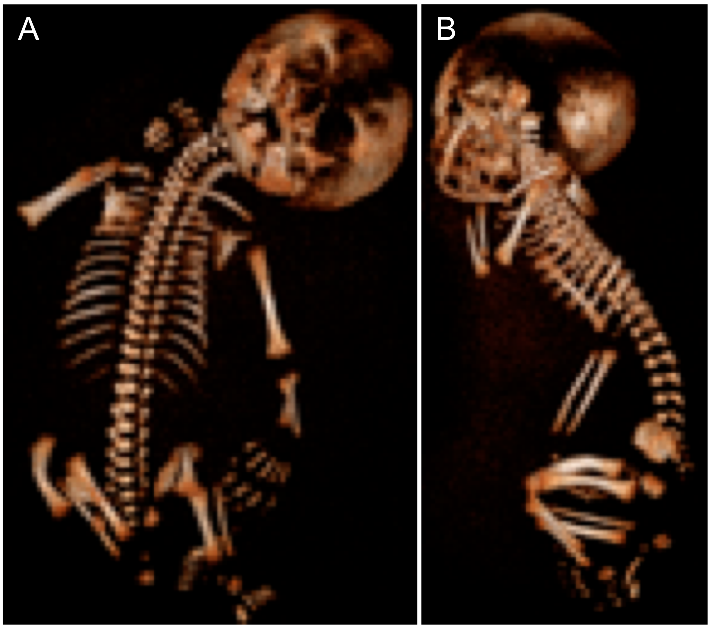

Supplementary Figure 4. Fetal CT (at unknown GA) of a fetus from family 4 with ATD and pathogenic variants in *DYNC2H1*. (A-B) CT shows short ribs, short ilia, mild shortening of the long bones and mild bowing of the femora.

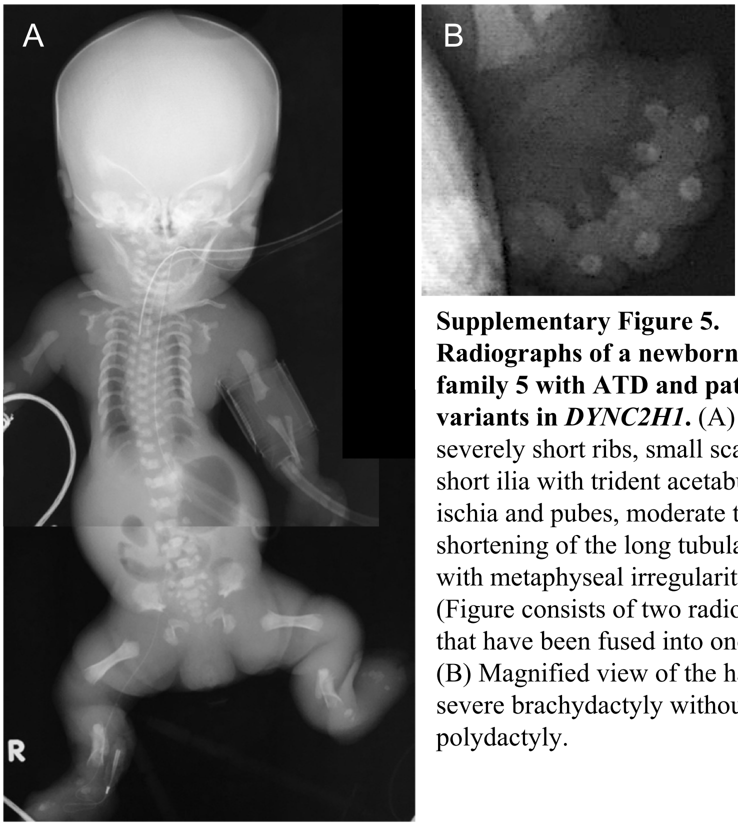

Supplementary Figure 5. Radiographs of a newborn from family 5 with ATD and pathogenic variants in *DYNC2H1*. (A) There are severely short ribs, small scapulae, short ilia with trident acetabula, short ischia and pubes, moderate to severe shortening of the long tubular bones with metaphyseal irregularities. (Figure consists of two radiographs that have been fused into one figure). (B) Magnified view of the hand shows severe brachydactyly without polydactyly.

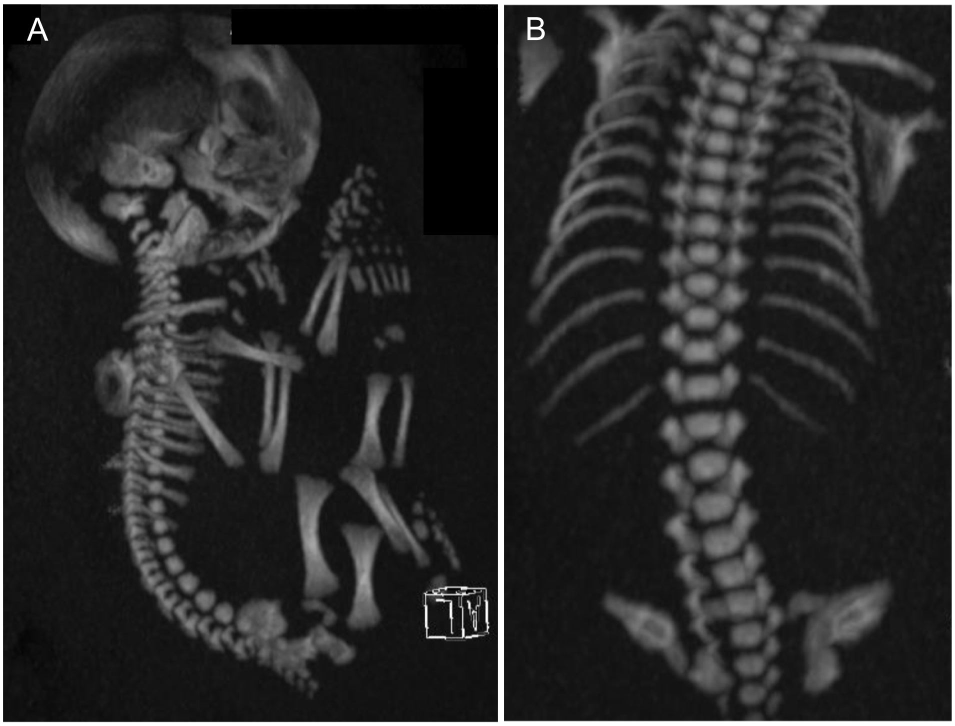

Supplementary Figure 6. Fetal CT (28 weeks GA) of proband from family 6 with ATD and pathogenic variants in *DYNC2H1*. (A-B) There are short ribs and mildly short long tubular bones with metaphyseal irregularities. The skull and spine are unremarkable.

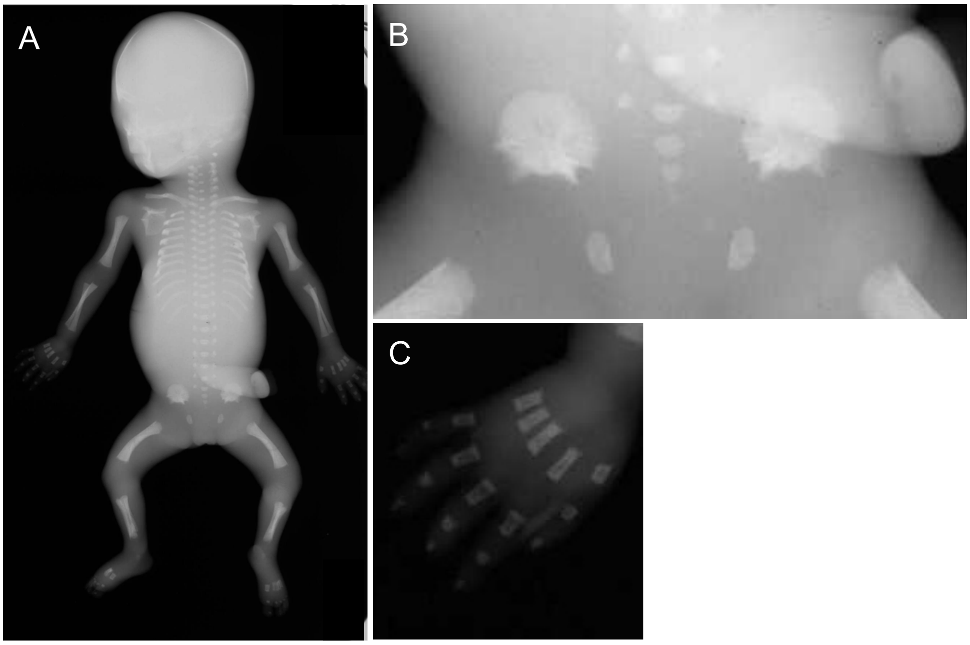

Supplementary Figure 7. Radiographs of a fetus from family 7 with ATD, a fetus TOP at 19 weeks GA, with pathogenic variants in *DYNC2H1*. (A) There are short ribs with splaying of the anterior ends, mild shortening of the long bones, and mild bowing of the femora. (B) Magnified view of the pelvis demonstrates short ilia with trident acetabula. (C) The hand shows mild brachydactyly.

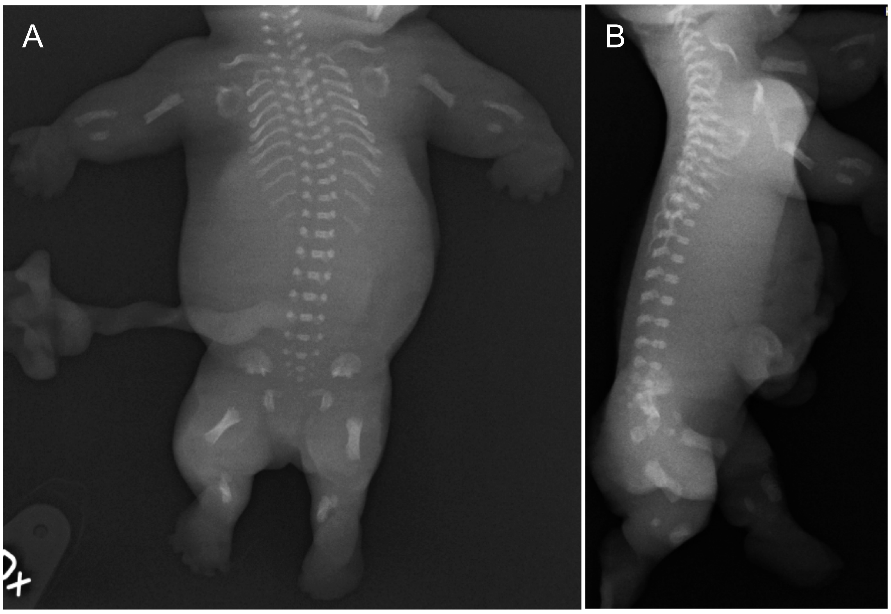

Supplementary Figure 8. Radiographs of a fetus from family 8 with SRPS3, TOP at 18 weeks GA, with pathogenic variants in *DYNC2H1*. (A-B) There are severely short ribs, short ilia with trident acetabula, and severe shortening of the long bones with metaphyseal irregularities. Mesomelic shortening is conspicuous with extremely short ulnae and fibulae. No ossification in hands and feet but the soft tissue are consistent with polydactyly.

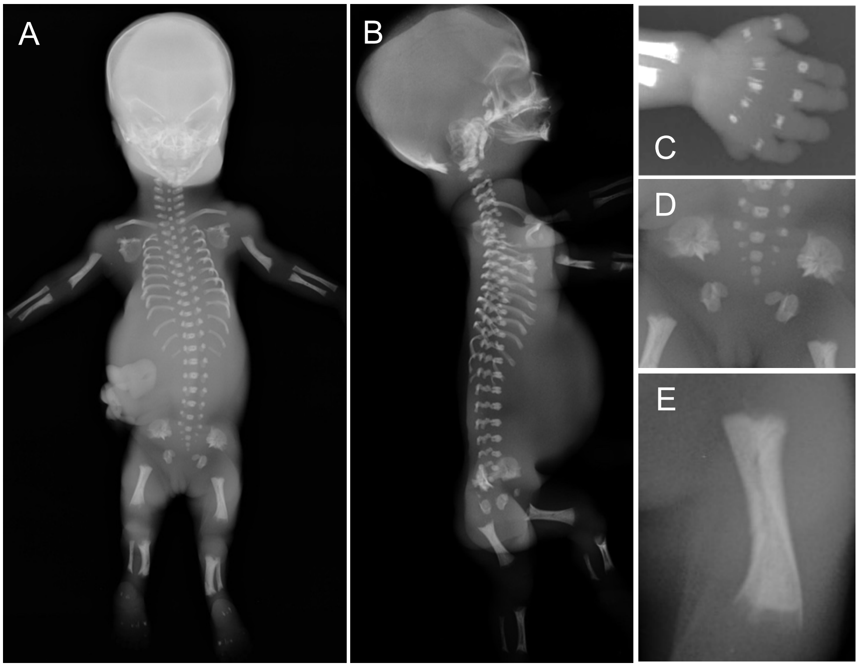

Supplementary Figure 9. Radiographs of fetus from family 9 with SRPS3, TOP at 21 weeks GA, with pathogenic variant in *DYNC2H1*. (A-E) There are short ribs, small scapulae, short ilia with trident acetabula, and moderate shortening of the long bones with metaphyseal irregularities. (C) Magnified left hand show severe brachydactyly and postaxial polydactyly.

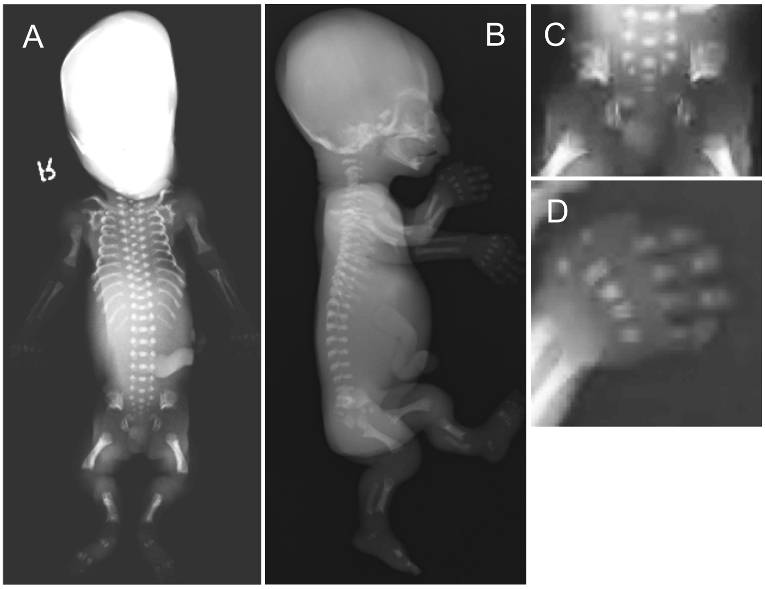

Supplementary Figure 10. Radiographs of fetus with ATD from family 10, TOP at 19 weeks GA, with pathogenic variants in *DYNC2H1*. (A-C) There are short ribs, short ilia with trident acetabula, moderate shortening of the long bones with mild metaphyseal irregularities, and mild bowing. (D) Magnified hand shows short metacarpals and brachydactyly.

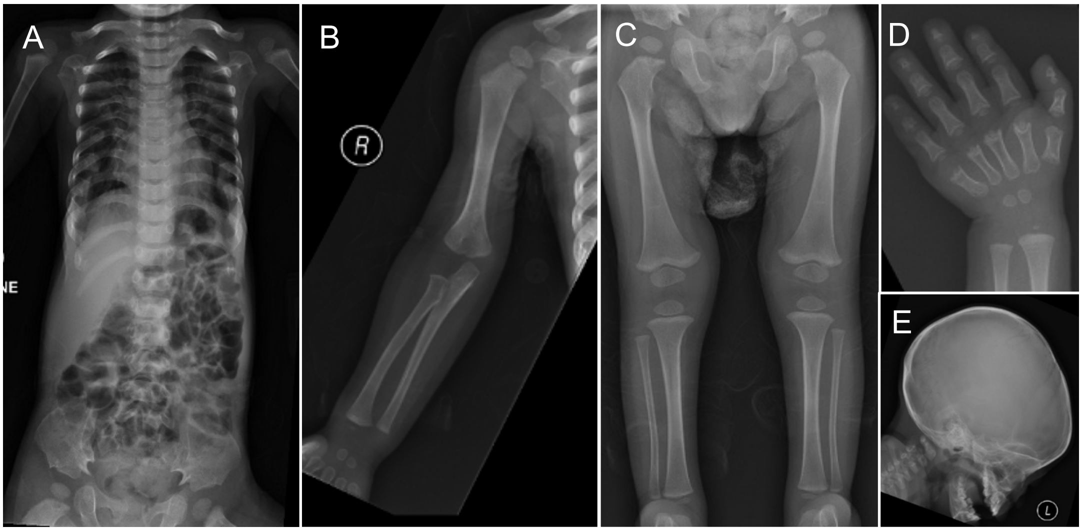

Supplementary Figure 11. Radiographs of a boy from family 11, 3 years of age, with ATD and pathogenic variants in *DYNC2H1*. (A) There are a handle-bar appearance of the clavicles, narrow thorax with short ribs, short ilia with trident acetabula. (B-C) Mild shortening of the long bones and round capital femoral epiphyses. (D) The hand shows brachydactyly and cone-shaped epiphyses. (E) The skull is unremarkable.

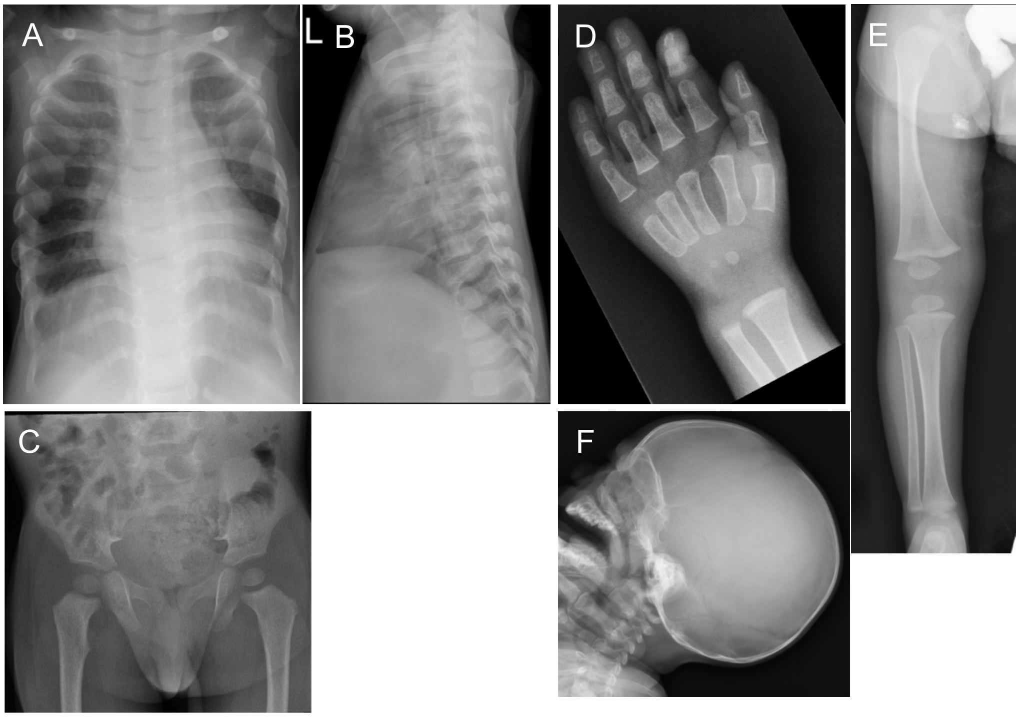

Supplementary Figure 12. Radiographs of a boy from family 12, with ATD and pathogenic variants in *DYNC2H1*. (A-B) At 17 months there is a narrow thorax and (C) mild hypoplasia of lower pelvis. (D) At 6 months mild brachydactyly and (E) mildly shortened long bones with mild femoral bowing and a subtle flare of the metaphyses is seen.

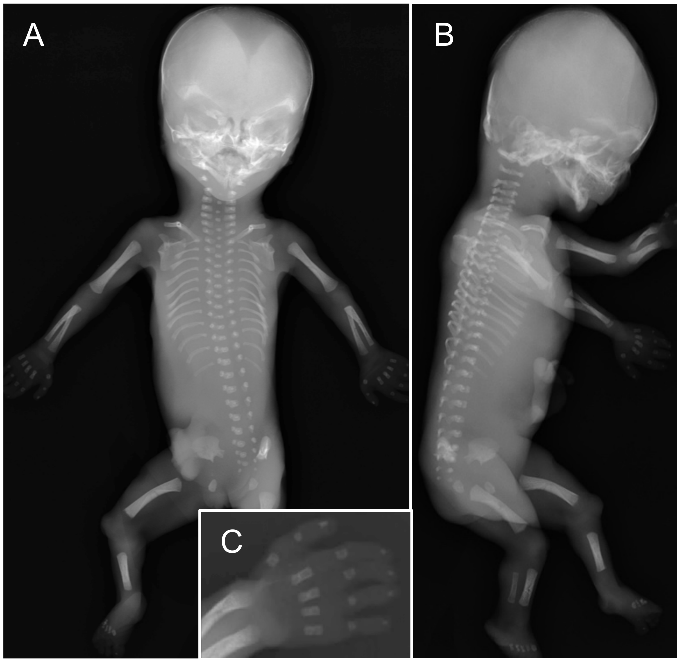

Supplementary Figure 13. Radiographs of a fetus from family 13 (TOP at 20 weeks GA) with pathogenic variants in *C2CD3*. (A-B) There are micrognathia, short ribs, short ilia and mild to moderate shortening of the long bones. (C) The hand shows mildly short metacarpals and phalanges.

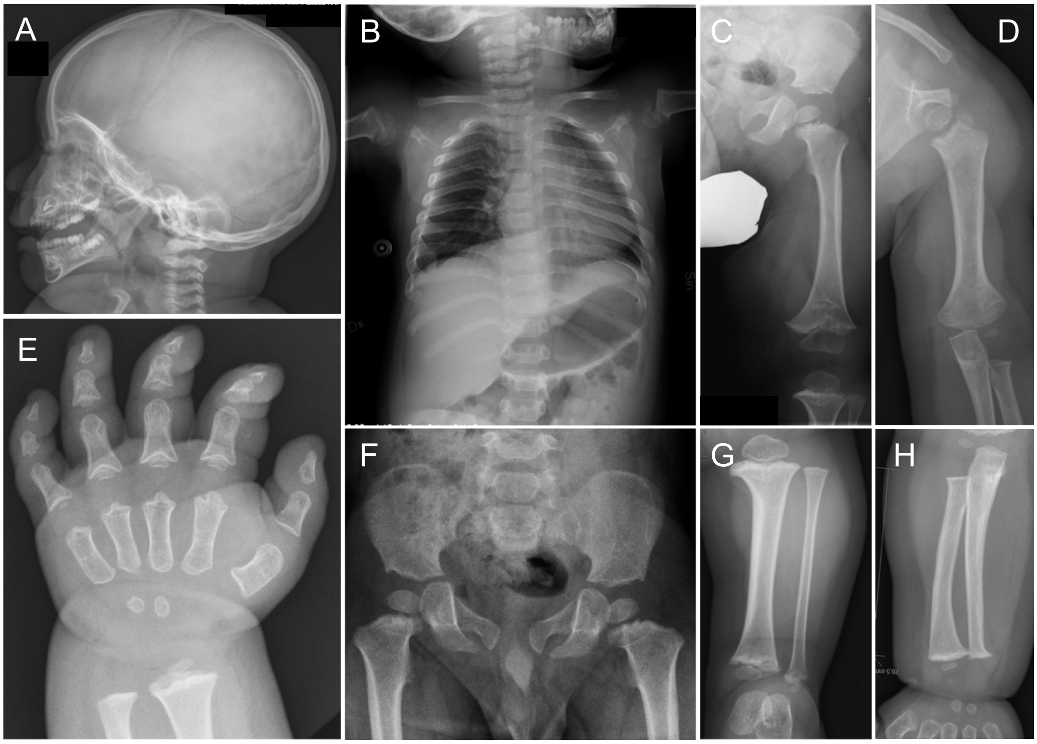

Supplementary Figure 14. Radiographs of a boy from family 16, 2 years of age, with ATD and pathogenic variants in *KIAA0753*. (A) The skull is unremarkable. (B) There are mildly short ribs. (C-D, G-H) Mild shortening of the long bones with metaphyseal irregularities. (E) Brachydactyly with cone-shaped epiphyses of the middle and proximal phalanges and the metacarpal bones. (F) Round capital femoral epiphyses and short trident ilia.

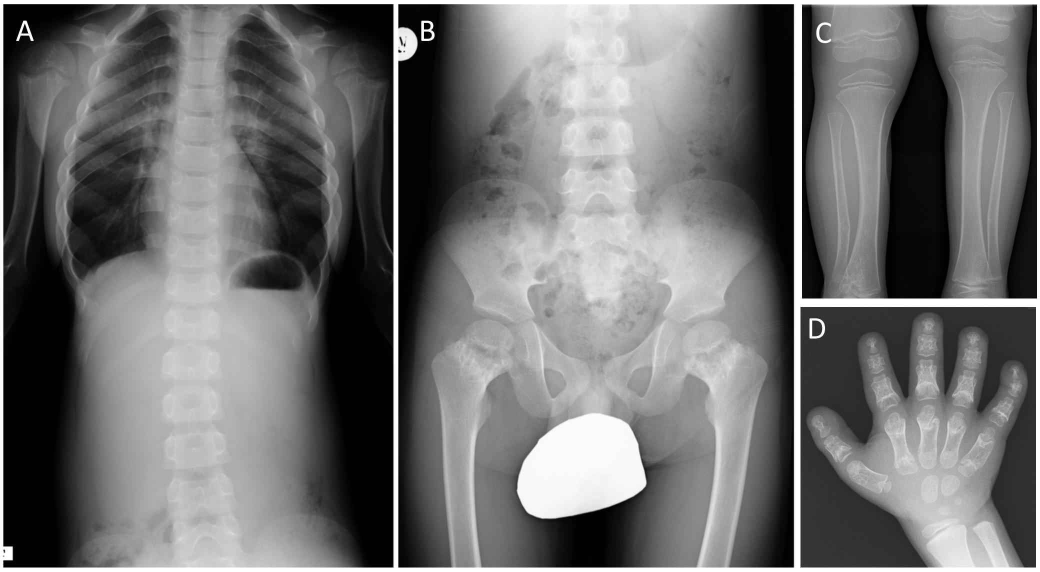

Supplementary Figure 15. Radiographs of a child from family 17, male with ATD and pathogenic variants in *TTC21B*. (A, B, C, D) short ribs, normal ilia, short and broad of the long tubular bones, striking metaphyseal irregularities of the proximal femora, brachydactyly with cone-shaped epiphyses and short metacarpals.

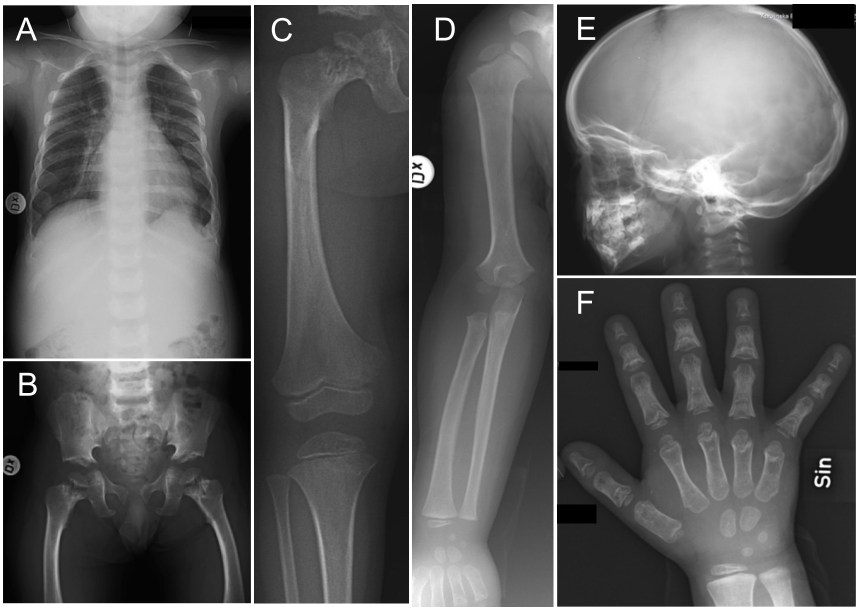

Supplementary Figure 16. Radiographs of patient 18, with pathogenic variants in *WDR19*, at the age of 4 years. (A) Note short ribs, (B) trident pelvis and (C) metaphyseal irregularities at the proximal femora. (C-D) There are mildly short tubular bones, with flared metaphyses. The femora is slightly bowed. (E) Dolichocephaly. (F) The hand shows cone-shaped epiphyses and mildly short metacarpals and phalanges.

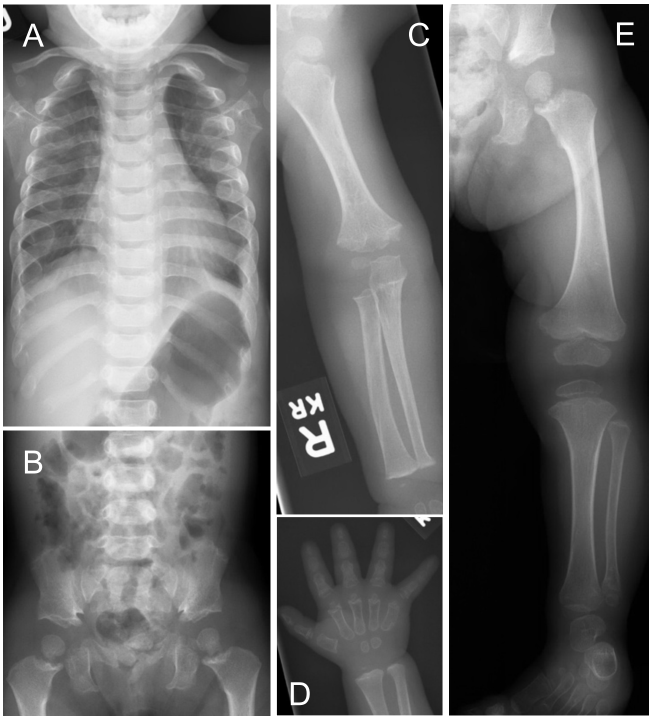

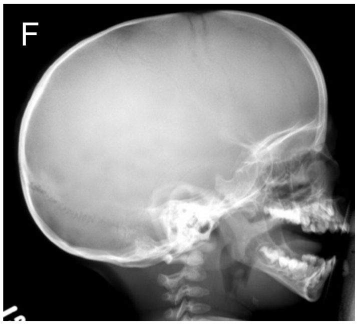

Supplementary Figure 17. Radiographs of a 2-year-old child from family 21 with ATD and pathogenic variants in *WDR19*. (A) The clavicles show a handle bar appearance, and the ribs are short with flared anterior ends. (B) Short greater sciatic notch, horizontal acetabula, and irregular ischia and pubes. (C and E) Mild shortening of the long tubular bones with flared and irregular metaphyses. (E) The proximal femora have rounded epiphyses. (D) The hand shows brachydactyly with cone-shaped epiphyses. (F) Dolichocephaly.

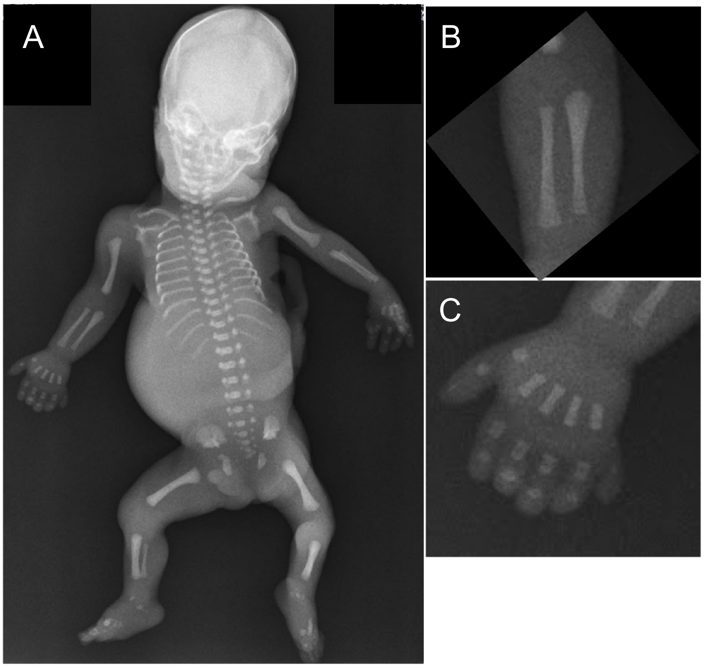

Supplementary Figure 18. Radiographs of a fetus from family 22 with pathogenic variants in EVC, TOP at 21 weeks GA. (A-B) Short ribs, short ilia with trident acetabula, and mild shortening of the long bones. The combination of bulbous ends of the the proximal ulna and distal radius with hypoplasia of the distal ulna and proximal radius gives rise to a chicken drumstick appearance of these bones. (C) Magnified view of the hand shows brachydactyly and postaxial polydactyly. (TOP, termination of pregnancy)

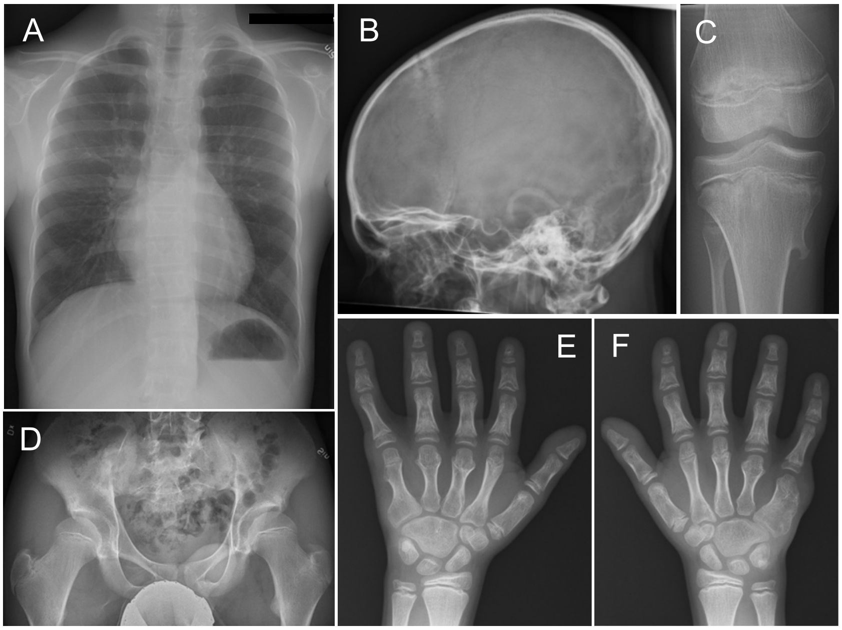

Supplementary Figure 19. Radiographs of a boy from family 23, age of 17 years, with pathogenic variants in *EVC2*. (A) Short ribs. (B) Normal skull. (C) Broad metaphyses and a small osteochondroma of the proximal tibia are noted. (D) Normal ilia and mildly broad femoral neck. (E-F) Severe brachydactyly with cone-shaped epiphyses especially of the middle phalanges. There is capitate-hamate synostosis. Surgically resected extra finger from right hand, postaxial. *Reused S19-figures from PMID:31965514*

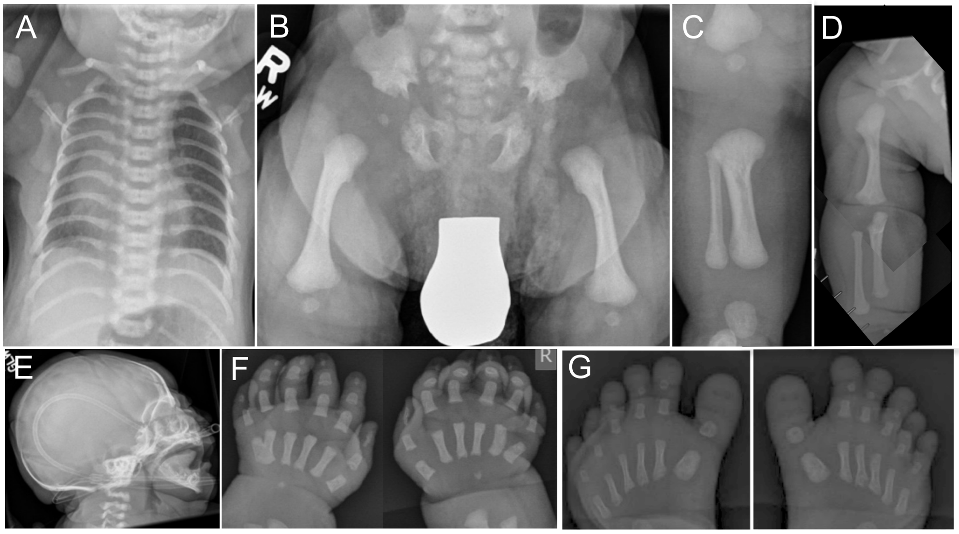

Supplementary Figure 20. Radiographs of a neonate from family 24, with clinical diagnosis of EVC, with pathogenic variant in *PRKACA*. (A) Narrow thorax with short ribs, (B) Short ilia with trident acetabula, (B-D) Short tubular bones with bulbous bone ends and a chicken drumstick appearance of the ulna and radius, as well as the tibia and fibula. Premature ossification of capital femoral epiphyses. (E) The skull is unremarkable. (F-G) The hands and feet show severe brachydactyly and postaxial polydactyly.

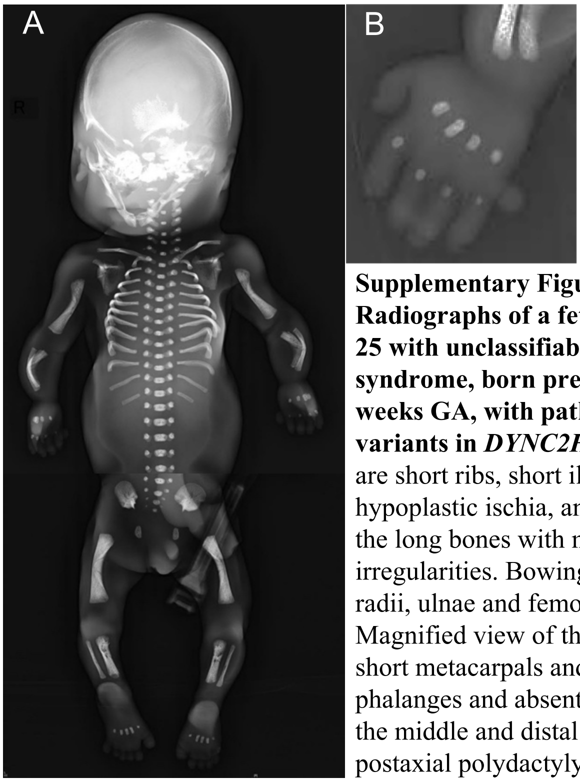

Supplementary Figure 21. Radiographs of a fetus from family 25 with unclassifiable short rib syndrome, born premature at 31 weeks GA, with pathogenic variants in *DYNC2H1*. (A) There are short ribs, short ilia, and hypoplastic ischia, and shortening of the long bones with mild metaphyseal irregularities. Bowing of the humeri, radii, ulnae and femora is seen. (B) Magnified view of the hand shows short metacarpals and proximal phalanges and absent ossification of the middle and distal phalanges, and postaxial polydactyly.

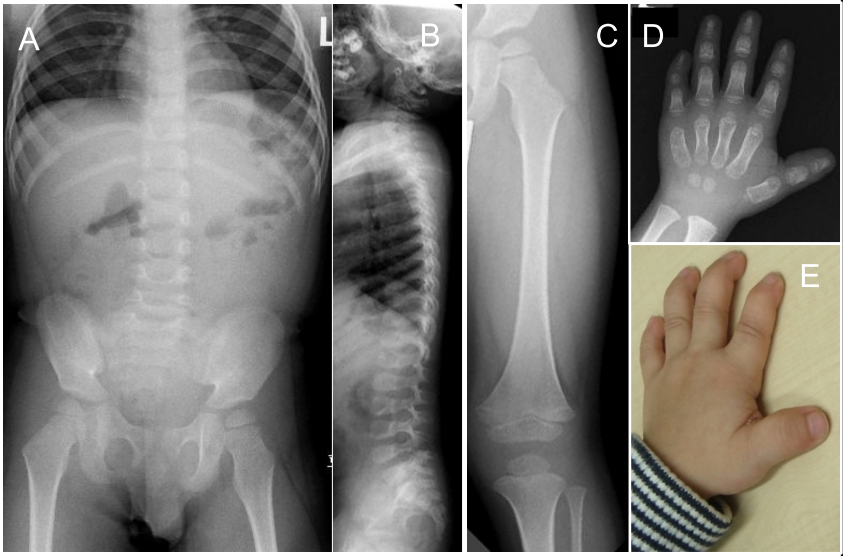

Supplementary Figure 22. Radiographs and clinical photograph of a child from family 26 with clinical diagnosis of mild skeletal ciliopathy at 2 years of age and molecular diagnosis of 1q24q25 microdeletion syndrome. (A, B, C) Normal chest, spine, pelvis, and long bones. (D-E) Brachydactyly with cone-shaped epiphyses particularly of the middle phalanges.

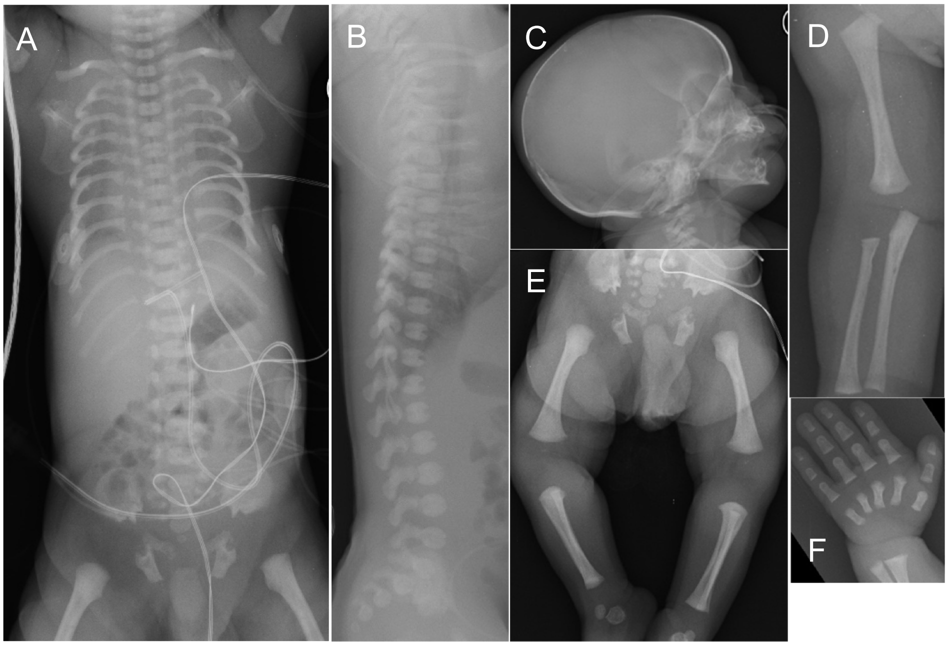

Supplementary figure 23. Radiographs of a neonate from family 27, with only one identified pathogenic variant in *DYNC2H1*. (A) There are short ribs with splaying of the anterior ends. (B-C) The skull and spine are unremarkable. (D-E) Short ilia with trident acetabula, hypoplastic pubes and ischia, and mild shortening of the long bones. (F) The hand shows short metacarpals.

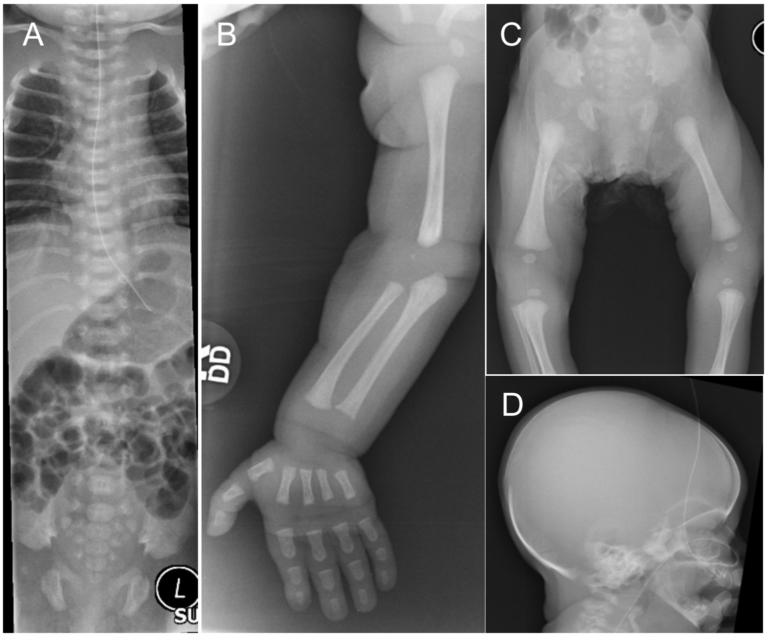

Supplementary Figure 24. Radiographs of a newborn from family 28, with ATD and unknown genetic etiology. (A-B) Short and gracile ribs, short ilia with trident acetabuli, and mild shortening of the long bones. The hand shows mild brachydactyly. (D) The skull is normal.

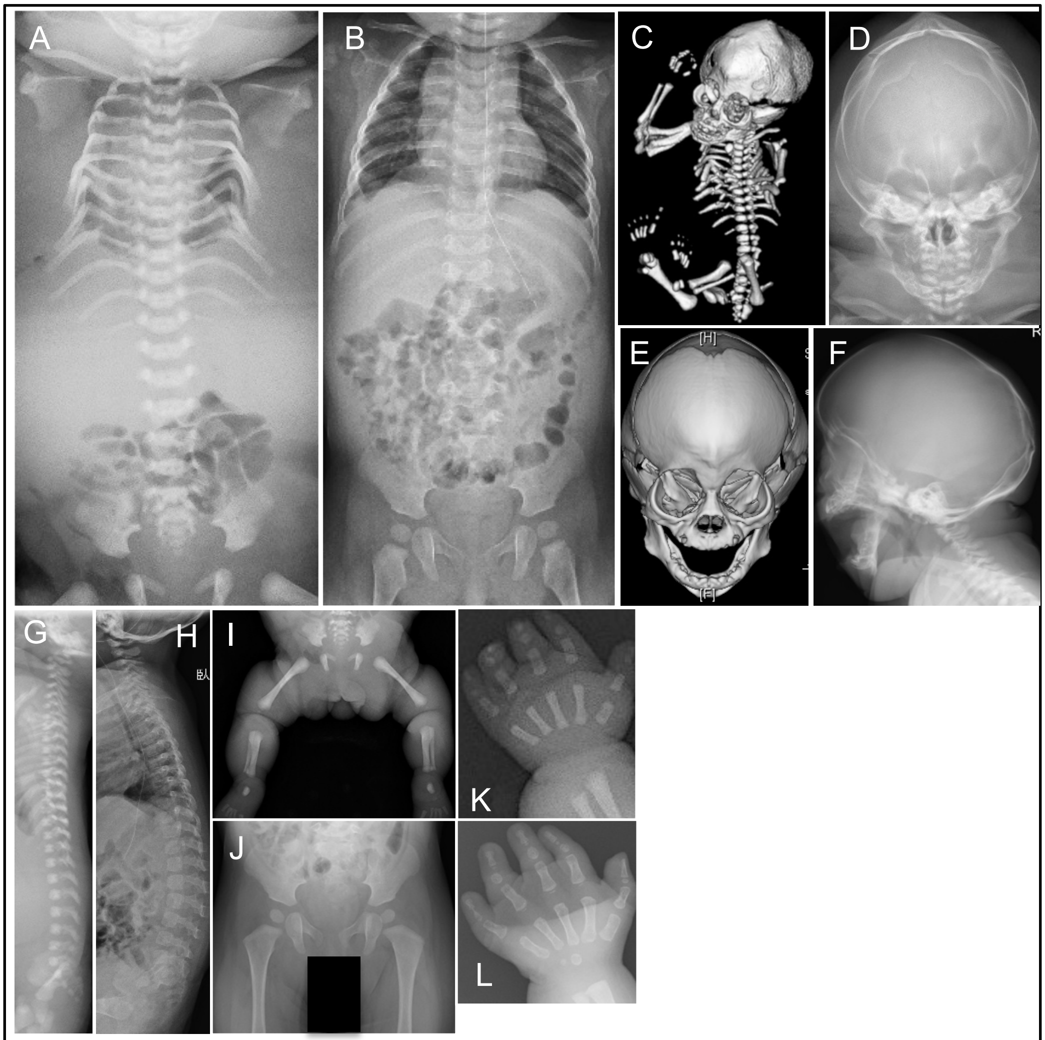

Supplementary Figure 25. Radiographs and fetal CT of two affected sibs from family 29 with Sensenbrenner syndrome, with only one candidate variant found in *IFT122*. (A) A newborn (male) (II:1) shows short and wavy ribs and (B) A child (female) (II-2) at 8 months of age shows mild thoracic hypoplasia. (C) Prenatal CT and (D) postnatal radiograph show turridolichocephaly (II:1). (E) Prenatal CT and (F) postnatal radiograph show premature fusion of the metopic and sagittal sutures (II:2). (G, II:1 and H, II:2) The spine shows mild dolichospondyly. (I) Mild shortening of the long bones with bulbous bone ends and normal pelvis (II:1). (J) Normal ilia, round capital femoral epiphyses (II:2). (K, II:1 and L, II:2) The hand shows short metacarpals and phalanges, particularly short middle phalanges. *S25-figures reused with permission from Japan Journal of Radiology, PMID:31965514*

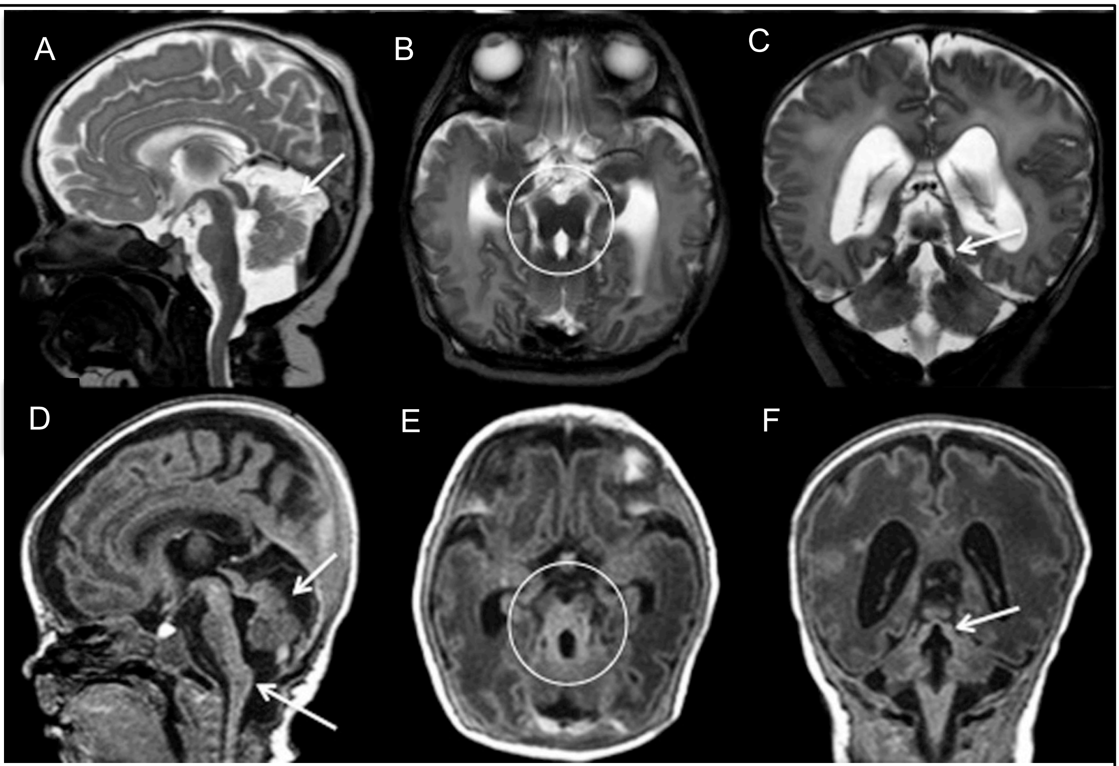

Supplementary Figure 26. MRI of two siblings from family 14 with features of Joubert syndrome and pathogenic variants in *KIAA0753*. (A-C) The first child, at 2 days and (D-F) the second child, at 4 days of life. (A) Sagittal T2W and (D) sagittal T1W images demonstrate hypoplastic cerebellar vermis with deficiency of the anterior lobes (upper arrows). Note the prominence of the clava (lower arrow) and poor brainstem transition in (D). (B) Axial T2W and (E) axial T1W images at the level of the midbrain reveal prominent superior cerebellar peduncles and the absence of their decussation, giving the characteristic "molar tooth" appearance (circles). (C) Coronal T2W and (F) coronal T1W images again show the thickened and elongated superior cerebellar peduncles (arrows).

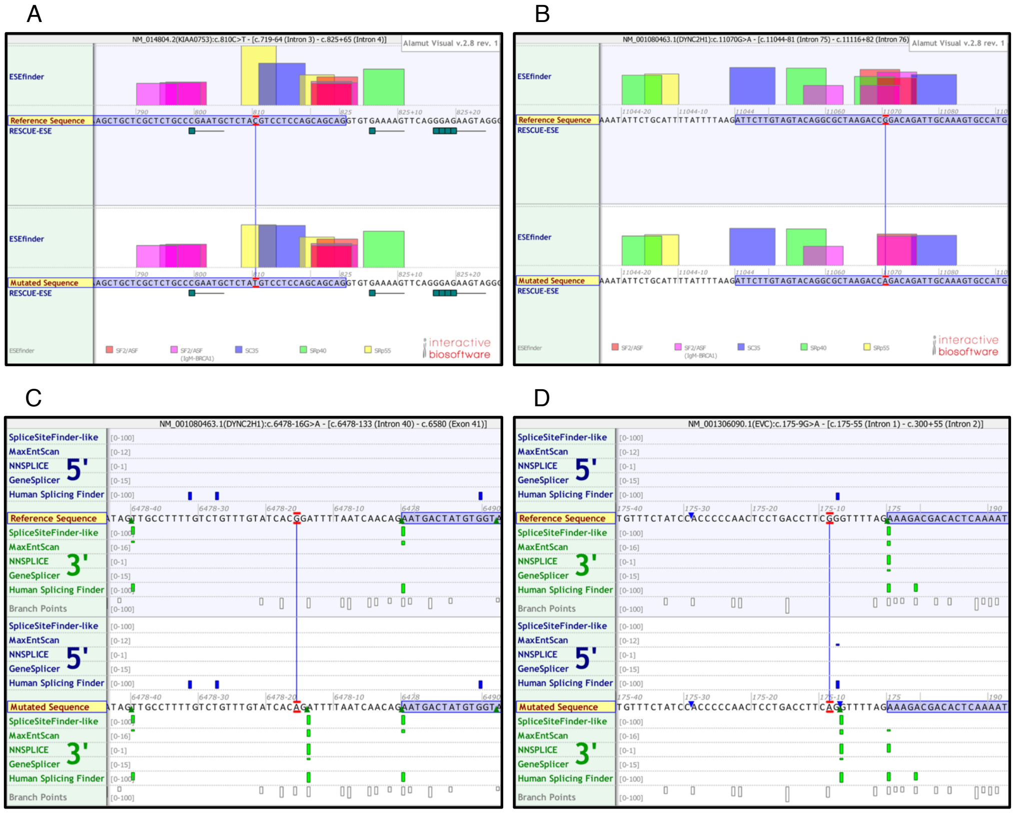

Supplemental Figure 27. Interpretation of splice variants using interactive-biosoftware in Alamut Visual v.2.8. (A) *KIAA0753* synonymous variant (c.810C>T) gives no convincing information about disruption of an ESE-element (using ESEfinder and RESCUE-ESE); (B) *DYNC2H1* synonymous variant (c.11070G>A) could be interpreted as loss of an ESE site according to ESEfinder, but hard to interpret. (C-D) *DYNC2H1* variant (c.6478-16G>A) and *EVC* variant (c.175-9G>A) gives a high probability that both variants introduce a cryptic splice site close to the canonical splice site. The blue line indicates were the pathogenic variant is located and the green bars indicate possible 3'splice sites according to different splice prediction softwares (SpliceSiteFinder-like, MaxEntScan, NNSPLICE, GeneSplicer and Human Splicing Finder). More information can be found: http://www.interactive-biosoftware.com/doc/alamut-visual/2.8/splicing.html

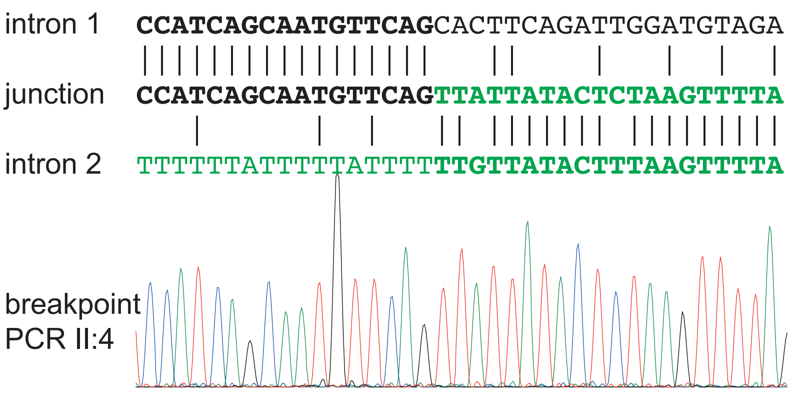

Supplementary Figure 28. Schematic presentation of the breakpoint junction of IFT74 between intron 1 and 2, and Sanger sequence trace of the breakpoint region from the affected child in family 20.
