# Supplementary material for: High diagnostic yield in skeletal ciliopathies using massively parallel genome sequencing, structural variant screening and RNA analyses
Source: J Hum Genet. 2021 Apr 20;66(10):995–1008. doi: 10.1038/s10038-021-00925-x (PMC8472897; doi:10.1038/s10038-021-00925-x)
Supplement: Supplementary file 2 — Supplementary Methods [file 10038_2021_925_MOESM2_ESM.pdf]

## 1.1 Bioinformatic platforms and MPS statistics

Three different analysis platforms were used, and variants were ranked for presence in coding exons ±20 base pairs intronic sequence and with a minor allele frequency of <0.005 according to public databases (1000G, dbSNP, ExAC/GnomAD) and our local in-house database. *In silico* prediction tools (PolyPhen-2 (http://genetics.bwh.harvard.edu/pph2), SIFT (http://sift.jcvi.org/), AlignGVGD (http://agvgd.hci.utah.edu/), MutationTaster (http://www.mutationtaster.org/)), amino acid conservation and variant segregation in the family were used to predict the pathogenicity of the variants. For variant interpretation and splice prediction we used Alamut Visual® (Interactive Biosoftware), ESE-finder (http://rulai.cshl.edu/) and RESCUE-ESE (http://genes.mit.edu).

## 1.2 MPS Pipelines (supplementary table 3)

Scout WGS as previously described (18)

Scout exome sequencing (ES) as previously described (17, 19), family

NGI ES as previously described (12).

OGT ES Exome sequencing (for family 2 and 21) using HiSeq2000 (Illumina) was performed with TruSeq v3 Chemistry at Oxford Gene Technology (OGT, Begbroke, UK). Reads were mapped to their location in the human genome (build hg19/b37) using Burrows-wheeler aligner (BWA, bio-bwa.sourceforge.net) package (version 0.6.2). Realignment of the mapped reads was carried out with GATK (version 1.6) and duplicate reads marked with Picard (version 1.107 broadinstitute.github.io/picard). SNP and indel variants were identified (Samtools 0.1.18, GATK unified genotyper). SNP novelty was annotated according to dbSNP release 135.
