# Supplementary material for: High diagnostic yield in skeletal ciliopathies using massively parallel genome sequencing, structural variant screening and RNA analyses
Source: J Hum Genet. 2021 Apr 20;66(10):995–1008. doi: 10.1038/s10038-021-00925-x (PMC8472897; doi:10.1038/s10038-021-00925-x)
Supplement: Supplementary file 3 — Supplementary Tables [file 10038_2021_925_MOESM3_ESM.pdf]

Supplementary Table 1. Short summary of the diagnostic criteria used in the study. Adapted from "Bone dysplasias" by Spranger et al, 4th edition.

	Short ribs	Polydactyly	Brachydactyly with cone-shaped epiphyses	Iliac hypoplasia & trident acetabua	Other Skeletal changes	Common extra-skeletal changes
**ATD Jeune**	+ ~++	Uncommon: postaxial (10%)	+ ~++	+	Metaphyseal irregularity of the proximal femora (occasionally)	Nephronophthisis<br>Hepatic fibrosis
**SRPS type 1/3**	+++	Common: postaxial	+++	+	Mild platyspondyly<br>Ragged metaphyses	Diverse malformations (brain, heart, liver, kidney, genitourinary)
**EVC**	+ ~++	Common: postaxial and mesoaxial	++ ~+++	+	Bulbous ends of tubular bones<br>Hypoplasis of the lateral portion of the proximal epiphysis with genu valgum<br>Lateral sloping of the humeri	Cardiac anomaly<br>Gum & teeth anomaly
**CED**	+ ~+++	Rare	+ ~++	–	Sagittal craniosynostosis<br>Posterior tibial bowing<br>Coat-hanger ribs	Sparse hair<br>Nephronophthisis<br>Hepatic fibrosis

ATD, asphyxiating thoracic dysotrophy; SRPS, short rib polydactly syndrome; CED, cranioectodermal dysplasia (also named Sensenbrenner syndrome)

Supplementary Table 2. Genes associated with SRTD, CED and EVC phenotypes and protein localization

Gene	Skeletal ciliopathy	Other clinical entities	Localization in cilia
C2CD3	SRTD<br>Cortes et al. (2016)	OFD14 [MIM:615948]	Basal body component
C21orf2	SRTD McInerney-Leo et al. (2017)	RDMS [MIM:617547]<br>SMDAX [MIM:602271]	Basal body component
CEP120	SRTD13 [MIM:616300]	JBTS31 [MIM:617761]	Basal body component
CSPP1	SRTD<br>Tuz et al. (2014)	JBTS21 [MIM:615636]	Basal body component
DYNC2H1	SRTD3 [MIM:613091]		IFT dynein subunit
DYNC2LI1	SRTD15 [MIM:617088]		IFT dynein subunit
EVC	EVC [MIM:225500]<br>WAD [MIM:193530]		INV compartment
EVC2	EVC [MIM:225500]<br>WAD [MIM:193530]		INV compartment
FUZ	SRTD Zhang et al. (2018)		CPLANE complex
ICK	SRTD Paige Taylor et al (2016)	ECO [MIM:612651]	IFT cilia regulators
IFT43	CED3 [MIM:614099]<br>SRTD18 [MIM:617866]	RP81 [617866]	Part of IFT-A
IFT52	SRTD16 [MIM:617102]		Part of IFT-B
*IFT74**		BBS20 [617119]	Part of IFT-B
IFT80	SRTD2 [MIM:611263]		Part of IFT-B
IFT81	SRTD19 [MIM:617895]		Part of IFT-B
IFT122	CED1 [MIM:218330]		Part of IFT-A
IFT140	SRTD9 [MIM:266920]	RP80 [MIM:617781]	Part of IFT-A
IFT172	SRTD10 [MIM:615630]	RP71 [MIM:616394]	Part of IFT-B
INTU	SRTD20 [MIM: 617926]	OFD17 [MIM:617926]	CPLANE complex,<br>Basal body component
KIAA0586	SRTD14 [MIM:616546]	JBTS23 [MIM:616490]	Basal body component
KIAA0753	SRTD with JBTS features Hammarsjö et al. (2017)	OFD15 [MIM:617127]	Centriolar satellites
LBR	SRTD Zhang et al. (2018)	GRBGD [MIM:215140]<br>PHA [MIM:169400]	Unclear
NEK1	SRTD6 [MIM:263520]	SMDAX Wang et al. 2017	Basal body component
NEK9	SRTD Casey et al. (2016)	LCCS10 [MIM:617022]	Unclear
TCTEX1D2	SRTD17 [MIM:617405]		IFT dynein subunit
TRAF3IP1	SRTD Zhang et al. (2018)	SLSN9 [MIM:616629]	Part of IFT-B<br>CPLANE complex
TTC21B	SRTD4 [MIM:613819]	NPHP12 [MIM:613820]	Part of IFT-A
WDR19	CED4 [MIM:614378]<br>SRTD5 [MIM:614376]	NPHP13 [MIM:614377]<br>SLSN8 [MIM:616307]	Part of IFT-A
WDR34	SRTD11 [MIM:615633]		IFT dynein subunit
WDR35	CED2 [MIM:613610]<br>SRTD7 [MIM:614091]		Part of IFT-A
WDR60	SRTD8 [MIM:615503]		IFT dynein subunit

* added to gene list after trio analysis of family 20

BBS, Bardet-Biedl syndrome; CED, Cranioectodermal dysplasia; CPLANE, ciliogenesis and planar polarity effector; ECO, Endocrine-cerebroosteodysplasia; EVC, Ellis-van Creveld; GRBGD, Greenberg dysplasia; IFT, Intraflagellar transport; INV, Inversin; JBTS, Joubert syndrome; LCCS, Lethal congenital contracture syndrome; MIM, Mendelian Inheritance in Man; NPHP, nephronophthisis; OFD, Orofaciodigital syndrome; RDMS, Retinal dystrophy with macular staphyloma; RP, Retinitis pigmentosa; SLSN, Senior-Loken syndrome; SMDAX, Spondylometaphyseal dysplasia, axial; SRTD, Short-rib thoracic dysplasia; WAD, Weyers acrofacial dysostosis

Supplementary Table 3. Sequencing statistics

Patient	Total reads [10^6]	Mean coverage [X]	Coverage [10X]	Coverage [20X]	MPS Pipeline*	CNV
**1**	98.8	178.3	99.1%	98.4%	Scout ES	a-CGH
**2**	29.2	67.2	97.5%	92.0%	OGT ES	a-CGH
**3**	84.0	265.1	99.4%	-	Scout ES	a-CGH
**4**	-	37.6	99.5%	96.5%	Scout WGS	SV WGS
**5**	70.7	135.0	98.0%	-	NGI ES	a-CGH
**6**	-	293.7	99.5%	-	Scout ES	a-CGH
**7**	-	39.2	99.3%	-	Scout WGS	SV WGS
**8**	-	35.9	99.4%	95.6%	Scout WGS	SV WGS
**9**	-	36.4	99.4%	98.9%	Scout WGS	SV WGS
**10**	-	37.7	99.4%	98.8%	Scout WGS	SV WGS
**11**	76.3	179.3	98.9%	98.1%	Scout ES	a-CGH
**12**	363.7	30.9	99.2%	92.8%	Scout WGS	SV WGS
**13**	-	34.7	99.3%	95.3%	Scout WGS	SV WGS
**14**	499.3	41.5	99.5%	99.3%	Scout WGS	SV WGS
**15**	-	38.4	99.4%	96.1%	Scout WGS	SV WGS
**16**	-	39.2	99.4%	96.3%	Scout WGS	SV WGS
**17**	292.5	27.1	98.8%	87.3%	Scout WGS	SV WGS
**18**	-	37.9	99.5%	99.3%	Scout WGS	SV WGS
**19**	-	36.0	99.5%	99.1%	Scout WGS	SV WGS
**20.1**	-	30.6	99.3%	92.7%	Scout WGS	SV WGS
**20.2 M**	-	29.8	99.7%	95.3%	Scout WGS	-
**20.3 P**	-	30.2	99.2%	96.5%	Scout WGS	-
**21**	29.7	77.9	98.0%	94.5%	OGT ES	a-CGH
**22**	-	34.5	99.5%	98.7%	Scout WGS	SV WGS
**23**	-	296.0	99.7%	-	Scout WES	a-CGH
**24.1**	-	32.1	99.4%	94.1%	Scout WGS	SV WGS
**24.2 M**	-	37.9	99.7%	99.4%	Scout WGS	-
**24.3 P**	-	36.4	99.6%	95.9%	Scout WGS	-
**25**	-	36.7	99.4%	95.8%	Scout WGS	SV WGS
**26**	-	39.4	99.1%	-	Scout WGS	a-CGH/SV
**27.1**	-	30.6	99.1%	96.4%	Scout WGS	a-CGH/SV
**27.2 M**	-	38.6	99.7%	99.5%	Scout WGS	-
**27.3 P**	-	35.1	99.6%	95.5%	Scout WGS	-
**28.1**	-	37.9	99.7%	99.4%	Scout WGS	SV WGS
**28.2 M**	-	35.7	99.7%	99.2%	Scout WGS	SV WGS
**28.3 P**	-	35.4	99.6%	95.6%	Scout WGS	SV WGS
**29.1**	-	34.7	99.7%	98.9%	Scout WGS	a-CGH/SV
**29.2 M**	-	48.7	94.7%	84.5%	Scout WGS	a-CGH/SV
**29.3 P**	-	49.3	94.8%	84.6%	Scout WGS	a-CGH/SV

*see explanation of pipelines in secion 1.2 in supplementary information
Based on the entire coding regions defined by HGNC of refGene hg19/build37
a-CGH, array comparative genome hybridization; CNV, Copy number variation; M, maternal; NGI, National Genomics Infrastructure; OGT, Oxford genome technology; P, paternal; S, affected sibling; SV, structural variant; ES, exome sequencing; WGS, whole genome sequencing

Supplementary Table 4. Radiographic features of patients

Family	Age & sex	Diagnosis	Craniosynostosis	Thoracic hypoplasia	Short ribs	Short ilia	Trident pelvis	Short tubular bones	Broad metaphyses	Bowed femora	Polydactyly	Brachydactyly	Additional information
1	† 2d F	ATD	-	+	+	+	+	+	+	-	-	+	
	† 8d M	ATD	-	+	+	+	+	+	+	-	-	+	
2	TOP M	ATD	-	+	+	+	+	+	+	+	-	+	Syndactyly of the hands
3	TOP M	SRPS3	-	+	+	+	+	+	+	+	+	+	Scoliosis, lumbar lordosis
4	2y M	ATD	-	+	+	+	+	+	+	+	-	+	
5	6y M	ATD	-	+	+	+	+	+	+	-	-	+	
6	TOP M	ATD	-	+	+	+	+	+	+	+	-	+	
7	TOP F	ATD	-	+	+	+	+	+	+	+	-	+	*Coxa vara*
8	TOP M	SRPS3	-	+	+	+	+	+	+	+	+	+	Syndactyly (hands and feet)
9	TOP F	SRPS3	+	+	+	+	+	+	+	+	+	+	
10	TOP M	ATD	-	+	+	+	+	+	+	+	-	+	
11	3y M	ATD	-	+	+	+	+	+	+	-	-	+	
12	3y M	ATD	-	+	+	+	+	+	+	+	-	+	
	† 2d M	ATD	-	+	+	+	+	+	na	na	-	+	
13	TOP M	SRPS3	-	+	+	+	+	+	-	+	-	+	Brachycephaly
14	† 2d M	ATD	-	+	+	+	+	+	-	-	-	+	
	† 4m F	ATD	-	+	+	+	+	+	-	-	-	+	
15	† 2m M	ATD + JBTS	-	+	+	+	+	+	+	-	-	+	
	TOP NA	ATD + JBTS	-	+	+	+	+	+	+	-	-	+	
16	2y M	ATD	-	+	+	+	+	+	-	-	-	+	
17	7y M	ATD	-	+	+	+	+	+	-	-	-	+	
18	18y M	ATD	+	+	+	-	+	+	+	+	-	+	
19	† 2y F	ATD	+	+	+	-	+	+	+	+	-	+	Dolichocephaly
20	† 7d M	ATD	-	+	+	+	+	+	-	-	-	+	Scaphocephaly
21	† 10m F	CED	+	+	+	-	-	+	-	-	-	+	Macrocephaly, dolichocephaly
22	TOP M	EVC	-	+	+	+	+	+	-	-	+	+	Syndactyly of left foot
23	17y3m M	EVC	-	+	+	+	na	+	-	-	+	+	
24	4y4m M	EVC	-	+	+	+	+	+	+	+	+	+	Camptodactyly of thumbs
25	† 1d M	unclassifiable	-	+	+	+	-	+	+	+	+	+	
26	5y M	unclassifiable	-	+	+	-	-	+	-	-	-	+	Brachycephaly
27	† 1d M	ATD	-	+	+	+	+	+	-	+	-	+	
29	3m F	ATD	-	+	+	+	+	+	-	-	-	+	
29	† 1d M	CED	+	+	+	-	-	+	-	-	-	+	
	2y4m F	CED	+	+	+	-	-	+	-	-	-	+	

†, diseased; +, present; -, absent; ATD, asphyxiating thoracic dystrophy; CED, Cranioectodermal dysplasia; d, days; EVC, Ellis-van Creveld syndrome; F, female; JBTS, Joubert syndrome; m, months; M, male; na, not available; TOP, termination of pregnancy; SRPS3, short-rib polydactyly syndrome type 3; y, years

Supplementary Table 5. Variants with genomic position and ACMG classification		
**Variant**	**Genomic position**	**ACMG**
NM_001080463.1(DYNC2H1):c.7919T>C	Chr11(GRCh37):g.103070036T>C	4
NM_001080463.1(DYNC2H1):c.9865G>A	Chr11(GRCh37):g.103114446G>A	4
NM_001080463.1(DYNC2H1):c.12602C>T	Chr11(GRCh37):g.103327017C>T	3
NM_001080463.1(DYNC2H1):c.8003T>G	Chr11(GRCh37):g.103070120T>G	3
NM_001080463.1(DYNC2H1):c.9044A>G	Chr11(GRCh37):g.103091449A>G	5
NM_001080463.1(DYNC2H1):c.6910G>A	Chr11(GRCh37):g.103058085G>A	4
NM_001080463.1(DYNC2H1):c.8444G>A	Chr11(GRCh37):g.103075683G>A	3
NM_001080463.1(DYNC2H1):c.5971A>T	Chr11(GRCh37):g.103048381A>T	3
NM_001080463.1(DYNC2H1):c.11284A>G	Chr11(GRCh37):g.103175330A>G	4
NM_001080463.1(DYNC2H1):c.5682_5683del	Chr11(GRCh37):g.103046971-103046972	5
NM_001080463.1(DYNC2H1):c.5771A>T	Chr11(GRCh37):g.103047060A>T	3
NM_001080463.1(DYNC2H1):c.729T>A	Chr11(GRCh37):g.102987406T>A	5
NM_001080463.1(DYNC2H1):c.1855C>T	Chr11(GRCh37):g.102996022C>T	5
NM_001080463.1(DYNC2H1):c.2386del	Chr11(GRCh37):g.103006489del	5
NM_001080463.1(DYNC2H1):c.10163C>T	Chr11(GRCh37):g.103124113C>T	4
NM_001080463.1(DYNC2H1):c.624_625delinsAA	Chr11(GRCh37):g.102987301-102987302	4
NM_001080463.1(DYNC2H1):c.2574+1G>A	Chr11(GRCh37):g.103006678G>A	5
NM_001080463.1(DYNC2H1):c.7129T>G	Chr11(GRCh37):g.103058304T>G	3
NM_001080463.1(DYNC2H1):c.6478-16G>A	Chr11(GRCh37):g.103055609G>A	4
NM_001080463.1(DYNC2H1):c.1306G>T	Chr11(GRCh37):g.102991711G>T	5
NM_001080463.1(DYNC2H1):c.11070G>A	Chr11(GRCh37):g.103158288G>A	4
NM_001080463.1(DYNC2H1):c.1366C>T	Chr11(GRCh37):g.102992106C>T	5
NM_001080463.1(DYNC2H1):c.3455T>C	Chr11(GRCh37):g.103025332T>C	3
NM_001080463.1(DYNC2H1):c.5690T>C	Chr11(GRCh37):g.103046979T>C	3
NM_001080463.1(DYNC2H1):c.8354C>A	Chr11(GRCh37):g.103075593C>A	3
NM_015531.6(C2CD3):c.5227G>T	Chr11(GRCh37):g.73760516C>A	3
NM_015531.6(C2CD3):c.5267G>A	Chr11(GRCh37):g.73760476C>T	3
NM_014804.3(KIAA0753):c.810C>T	Chr17(GRCh37):g.6528090G>A	5
NM_014804.3(KIAA0753):c.970C>T	Chr17(GRCh37):g.6526336G>A	5
NM_024753.5(TTC21B):c.2758-2A>G	Chr2(GRCh37):g.166756392T>C	5
NM_024753.5(TTC21B):c.3857T>C	Chr2(GRCh37):g.166732691A>G	3
NM_025132.4(WDR19):c.56T>G	Chr4(GRCh37):g.39187395T>G	4
NM_025132.4(WDR19):c.3868_3871del	Chr4(GRCh37):g.39279778-39279781	5
NM_025132.4(WDR19):c.974T>C	Chr4(GRCh37):g.39217473T>C	3
NM_025132.4(WDR19):c.3758G>A	Chr4(GRCh37):g.39278681G>A	3
NM_025132.4(WDR19):c.1623C>G	Chr4(GRCh37):g.39226647C>G	5
NM_025132.4(WDR19):c.3533G>A	Chr4(GRCh37):g.39274649G>A	5
NM_001306090.1(EVC):c.1018C>T	Chr4(GRCh37):g.5749953C>T	5
NM_001306090.1(EVC):c.175-9G>A	Chr4(GRCh37):g.5720966G>A	4
NM_147127.5(EVC2):c.571A>G	Chr4(GRCh37):g.5691019T>C	3

**Continued: Supplementary Table 6. Variants with genomic position and ACMG classification**		
**Variant**	**Genomic position**	**ACMG**
NM_002730.4(PRKACA):c.409G>A	Chr19(GRCh37):g.14211648C>T	5
NM_001080463.1(DYNC2H1):c.10022C>G	Chr11(GRCh37):g.103116062C>G	3
NM_025103.3(IFT74)	Chr9(GRCh37):g.26959922-26962969	4
NM_001080463.1(DYNC2H1)	Chr11(GRCh37):g.103016481-103177263	5
NC_000001.11(1q24q25)	Chr1(GRCh37):g.169095250-175778910	5

Supplementary Table 6. Candidate variants for the unsolved probands after filtering WGS-trio data (GRCh37)

Family	Gene	Refseq	Nucleotide	Amino acid	Inheritance	CADD	Genomic coordinates	Gene OMIM	Comments
27	***DYNC2H1***	**NM_001080463.2**	**c.1540C>T**	**p.(Arg514*)**	**Paternal**	**37**	**11:102993608**	**603297**	**No plausible compound**
	FN3KRP	NM_024619.4	c.199dup	p.(Thr67Asnfs*47)	Homozygous	29	17:80676838		MAF in SAS pop gnomAD 0.4%
	ADGRE2	NM_001271052	c.1416+3G>C	p.(?)	Paternal	16	19:14866463	606100	
			c.781+5G>A	p.(?)	Maternal	4	19:14876465	606100	
	B9D1	NM_015681.6	c.467G>C	p.(Arg156Pro)	Maternal	30	17:19247108	614144	No plausible compound
	PKN3	NM_013355.5	c.779C>T	p.(Ala260Val)	de novo	18	9:131469628		Only 6/31 reads
	PKHD1	NM_138694.4	c.11377G>T	p.(Ala3793Ser)	de novo	0	6:51512850	606702	Known in AR disease
28	***KIAA0586***	**NM_014749**	**c.3170del**	**p.(Leu1057Cysfs*9)**	**Maternal**	**26**	**14:58954695**	**610178**	**No plausible compound**
	ADAP1	NM_006869	c.580G>A	p.(Gly194Ser)	*de novo*	25	7:943831		
	APOB	NM_000384.3	c.4838G>C	p.(Ser1613Thr)	Paternal	13	2:21234902	107730	
			c.10427C>A	p.(Ala3476Glu)	Maternal	0	2:21229313	107730	
29	***TTC21B***	**NM_024753.5**	**c.2569G>A**	**p.(Ala857Thr)**	**Maternal**	**33**	**2:166758420**	**612014**	**No plausible compound**
	IFT122	**NM_018262.4**	**c.1306G>A**	**p.(Gly436Arg)**	**Paternal**	**32**	**3:129198760**	**606045**	**No plausible compound; PMID: 23826986**
	PIEZO1	NM_001142864.4	c.6592C>T	p.(Arg2198Cys)	*de novo*	26	16:88783499	611184	
	KCTD17	NM_024681.3	c.834del	p.(Lys278Asnfs*69)	*de novo*	33	22:37458573	616386	Only 5/30 reads
	NOS2	NM_000625	c.595C>T	p.(Arg199Cys)	Paternal	27	17:26110005	163730	
			c.1509C>A	p.(Asp503Glu)	Maternal	7	17:26100237	163730	
	HMCN1	NM_031935.3	c.5706C>T	p.(Asn1902=)	Maternal	9	1:185992242	608548	
			c.7955C>T	p.(Ala2652Val)	Paternal	26	1:186038870	608548	

Trio-analysis against all genes GRCh37 with autosomal dominant (de novo) inheritance, autosomal recessive inheritance (compound heterozygous or homozygous) and X-linked inheritance. Variants in known cilia genes with autosomal recessive inheritance when no plausible compound was found is marked as bold.
